# Supplementary material for: Dynamics of B cell repertoires and emergence of cross-reactive responses in patients with different severities of COVID-19
Source: Cell Rep. 2021 May 9;35(8):109173. doi: 10.1016/j.celrep.2021.109173 (PMC8106887; doi:10.1016/j.celrep.2021.109173)
Supplement: Document S2. Article plus supplemental information [file mmc5.pdf]

Cell Reports

Article

Dynamics of B cell repertoires and emergence of cross-reactive responses in patients with different severities of COVID-19

Graphical abstract

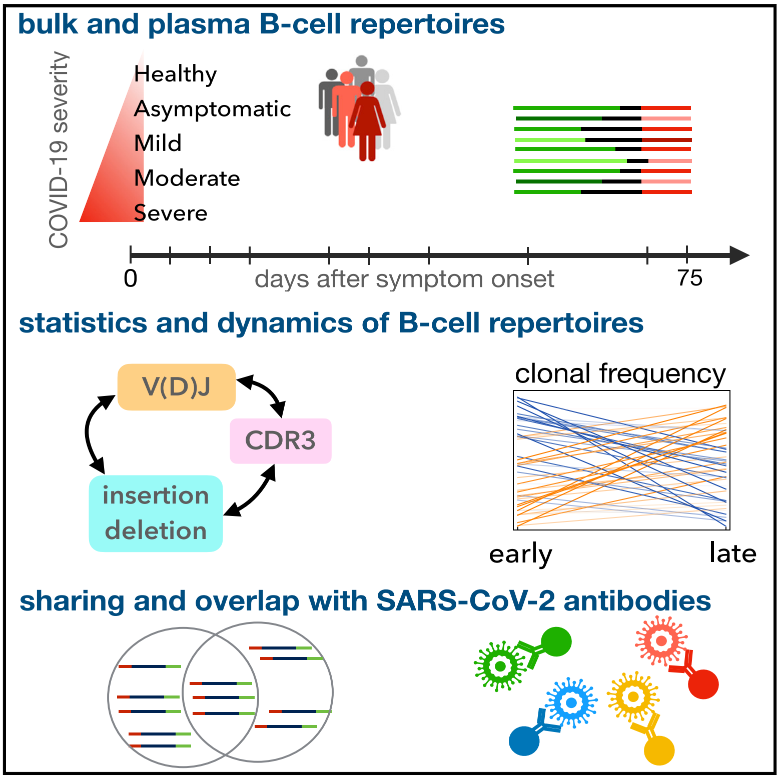

Highlights

- Analysis of B cell repertoires with SARS-CoV-2 epitope-sorted B cell receptors
- Differential sequence features of B cell receptors are associated with disease severity
- Expansion of B cell clonal lineages in response to SARS-CoV-2
- Shared B cell receptors emerge with cross-reactivity to SARS-CoV-1 and SARS-CoV-2

Authors

Zachary Montague, Huibin Lv, Jakub Otwinowski, ..., Nicholas C. Wu, Armita Nourmohammad, Chris Ka Pun Mok

Correspondence

nicwu@illinois.edu (N.C.W.), armita@uw.edu (A.N.), kapunmok@cuhk.edu.hk (C.K.P.M.)

In brief

It is unclear how the dynamics of the humoral immune response to SARS-CoV-2 vary across individuals with different disease severity. Montague et al. develop a principled statistical approach based on time-course, high-throughput B cell repertoire sequences to identify shared, expanding, rare clonal B cell lineages as candidates for responses specific to SARS-CoV-2.

Montague et al., 2021, Cell Reports *35*, 109173
May 25, 2021 © 2021 The Author(s).
https://doi.org/10.1016/j.celrep.2021.109173

CellPress

Cell Reports

CellPress
OPEN ACCESS

Article

Dynamics of B cell repertoires and emergence of cross-reactive responses in patients with different severities of COVID-19

Zachary Montague,[1,15] Huibin Lv,[2,15] Jakub Otwinowski,[3,17] William S. DeWitt,[4,5] Giulio Isacchini,[3,6] Garrick K. Yip,[2] Wilson W. Ng,[2] Owen Tak-Yin Tsang,[7] Meng Yuan,[8] Hejun Liu,[8] Ian A. Wilson,[8,9] J.S. Malik Peiris,[2] Nicholas C. Wu,[10,11,12,16,*] Armita Nourmohammad,[1,3,5,16,18,*] and Chris Ka Pun Mok[2,13,14,16,*]

[1]Department of Physics, University of Washington, 3910 15th Ave. Northeast, Seattle, WA 98195, USA
[2]HKU-Pasteur Research Pole, School of Public Health, Li Ka Shing Faculty of Medicine, The University of Hong Kong, Hong Kong SAR, China
[3]Max Planck Institute for Dynamics and Self-Organization, Am Faßberg 17, 37077 Göttingen, Germany
[4]Department of Genome Sciences, University of Washington, 3720 15th Ave. NE, Seattle, WA 98195, USA
[5]Fred Hutchinson Cancer Research Center, 1100 Fairview Ave. N, Seattle, WA 98109, USA
[6]Laboratoire de physique de l'ecole normale supérieure (PSL University), CNRS, Sorbonne Université, and Université de Paris, 75005 Paris, France
[7]Infectious Diseases Centre, Princess Margaret Hospital, Hospital Authority of Hong Kong, Hong Kong SAR, China
[8]Department of Integrative Structural and Computational Biology, The Scripps Research Institute, La Jolla, CA 92037, USA
[9]The Skaggs Institute for Chemical Biology, The Scripps Research Institute, La Jolla, CA 92037, USA
[10]Department of Biochemistry, University of Illinois at Urbana-Champaign, Urbana, IL 61801, USA
[11]Carl R. Woese Institute for Genomic Biology, University of Illinois at Urbana-Champaign, Urbana, IL 61801, USA
[12]Center for Biophysics and Quantitative Biology, University of Illinois at Urbana-Champaign, Urbana, IL 61801, USA
[13]Li Ka Shing Institute of Health Sciences, Faculty of Medicine, The Chinese University of Hong Kong, Shatin, Hong Kong SAR, China
[14]The Jockey Club School of Public Health and Primary Care, The Chinese University of Hong Kong, Hong Kong SAR, China
[15]These authors contributed equally
[16]These authors contributed equally
[17]Present address: Dyno Therapeutics, 1 Kendall Square, Building 1400E, Suite 202, Cambridge, MA 02139, USA
[18]Lead contact
*Correspondence: nicwu@illinois.edu (N.C.W.), armita@uw.edu (A.N.), kapunmok@cuhk.edu.hk (C.K.P.M.)
https://doi.org/10.1016/j.celrep.2021.109173

SUMMARY

Individuals with the 2019 coronavirus disease (COVID-19) show varying severity of the disease, ranging from asymptomatic to requiring intensive care. Although monoclonal antibodies specific to the severe acute respiratory syndrome coronavirus 2 (SARS-CoV-2) have been identified, we still lack an understanding of the overall landscape of B cell receptor (BCR) repertoires in individuals with COVID-19. We use high-throughput sequencing of bulk and plasma B cells collected at multiple time points during infection to characterize signatures of the B cell response to SARS-CoV-2 in 19 individuals. Using principled statistical approaches, we associate differential features of BCRs with different disease severity. We identify 38 significantly expanded clonal lineages shared among individuals as candidates for responses specific to SARS-CoV-2. Using single-cell sequencing, we verify the reactivity of BCRs shared among individuals to SARS-CoV-2 epitopes. Moreover, we identify the natural emergence of a BCR with cross-reactivity to SARS-CoV-1 and SARS-CoV-2 in some individuals. Our results provide insights important for development of rational therapies and vaccines against COVID-19.

INTRODUCTION

The novel severe acute respiratory syndrome coronavirus 2 (SARS-CoV-2), which causes the 2019 coronavirus disease (COVID-19), has now spread to 223 countries and caused more than 143 million infections with a mortality rate around 2.2% (World Health Organization, 2021). Individuals with COVID-19 show varying disease severity, ranging from asymptomatic to requiring intensive care. Although epidemiological and clinical data report that many factors, such as age, gender, genetic background, and pre-existing conditions, are associated with disease severity, host immunity against virus infection is the crucial component of controlling disease progression (Ellinghaus et al., 2020; Guan et al., 2020; McKechnie and Blish, 2020; Vabret et al., 2020; Wu et al., 2020a). Shedding light on signatures of a protective immune response against SARS-CoV-2 infection can help elucidate the nature of COVID-19 and guide therapeutic agent development as well as vaccine design and assessment.

Adaptive immunity is considered one of the core protective mechanisms of humans against infectious diseases. A vast

This is an open access article under the CC BY license (http://creativecommons.org/licenses/by/4.0/).

diversity of surface receptors on B and T cells enables us to recognize and counter new or repeated invasion from a multitude of pathogens (Janeway et al., 2005; Nielsen and Boyd, 2018). In particular, antibodies produced by B cells can provide long-lasting protection against specific pathogens through neutralization or other antibody-mediated immune mechanisms (Janeway et al., 2005). During the early phase of an infection, antigens of a pathogen are recognized by a group of naive B cells, which then undergo affinity maturation in a germinal center through somatic hypermutation and selection. The B cell receptors (BCRs) of mature B cells can react strongly to infecting antigens, resulting in B cell stimulation, clonal expansion, and, ultimately, secretion of high-affinity antibodies in the blood (Burnet, 1959, 1960; Cyster and Allen, 2019). The specificity of a BCR is determined by a number of features, such as V, D, or J gene usage and length and sequence composition of the HCDR3 region. SARS-CoV-2-specific immunoglobulin G (IgG) antibodies can be detected in plasma samples of individuals with COVID-19 starting from the first week after symptom onset (Perera et al., 2020). These antibodies bind to different antigens, including the spike protein and nucleoprotein as well as other structural or non-structural proteins (Hachim et al., 2020). In addition, multiple studies have isolated SARS-CoV-2-specific B cells from individuals with COVID-19 and determined their germline origin (Barnes et al., 2020; Brouwer et al., 2020; Cao et al., 2020; Chi et al., 2020; Han et al., 2020; Hansen et al., 2020; Hurlburt et al., 2020; Ju et al., 2020; Kreer et al., 2020a; Kreye et al., 2020; Liu et al., 2020b; Noy-Porat et al., 2020; Robbiani et al., 2020; Rogers et al., 2020; Seydoux et al., 2020a, 2020b; Shi et al., 2020; Wu et al., 2020b; Yuan et al., 2020; Zost et al., 2020). However, we still lack a comprehensive view of individuals' entire BCR repertoires during SARS-CoV-2 infection.

Antibody repertoire sequencing has advanced our understanding of the diversity of adaptive immune repertoires and their response to pathogens (Boyd et al., 2009; Georgiou et al., 2014; Kreer et al., 2020b; Robins, 2013). A few studies have performed BCR repertoire bulk sequencing to characterize the statistical signatures of the immune response to SARS-CoV-2 (Galson et al., 2020; Nielsen et al., 2020; Niu et al., 2020; Schultheiß et al., 2020). However, these studies have limited data regarding the dynamics of BCR repertoires, which could provide significant insight into responses specific to the infection. Moreover, they do not probe the composition of plasma B cells during infection, which is the direct indicator of antibody production in an individual.

In this study, we established a principled statistical approach to study the statistics and dynamics of bulk and plasma B cell repertoires and to characterize the immune responses in 19 individuals with different severities of COVID-19. By combining information from the statistics of sequence features in BCR repertoires, the expanding dynamics of clonal lineages during infection, and sharing of BCRs among individuals with COVID-19, we identified 38 clonal lineages that are potential candidates for a response to SARS-CoV-2. Importantly, eight of these lineages contain BCRs from the plasma B cell repertoire and, hence, are likely to have been secreting antibodies during infection. Moreover, using single-cell sequencing, we verified the reactivity of BCRs shared among individuals to the epitopes of the receptor-binding domain (RBD) and N-terminal domain (NTD) of SARS-CoV-2. Last, we identified cross-reactive responses to SARS-CoV-1 in some individuals with COVID-19 and a natural emergence of a previously isolated SARS-reactive antibody (Pinto et al., 2020) in three individuals.

RESULTS

Strong correlation between composition of bulk and plasma B cell repertoires

We obtained total RNA from peripheral blood mononuclear cells (PBMCs) isolated from 19 individuals infected with SARS-CoV-2 and three healthy individuals (STAR Methods; Data S1; Table S1). To broaden our healthy control pool, we also incorporated into our analyses IgG B cells from 10 individuals in the Great Repertoire Project (GRP) (Briney et al., 2019). Sequence statistics for the first three biological replicates pooled together for each individual from the GRP are shown in Data S1 (STAR Methods). The individuals with COVID-19 showed different severities of symptoms, forming three categories of infected cohorts: 2 individuals with mild symptoms, 12 with moderate symptoms, and 5 with severe symptoms. Specimens from all but one individual were collected at two or more time points during the course of the infection (Data S1). In addition to the bulk repertoire, we also isolated CD38^{+} plasma B cells from PBMC samples at at least two time points from seven individuals in this cohort (six moderate and one severe) and from seven additional individuals (two asymptomatic, three mild, and two moderate) and three healthy individuals (Figure S1; Data S1). The sampled time points for all individuals in this study are indicated in Figure 1 and Data S1. IgG heavy chains of B cell repertoires were sequenced by next-generation sequencing, and the statistics of the collected BCR read data from each sample are shown in Data S1. Statistical models were applied to analyze the length of the HCDR3 region, IGHV or IGHJ gene usage, and expansion and sharing of clonal lineages (Figure 1).

The bulk repertoire is a collection of all BCRs circulating in the blood, including receptors from naive, memory, and plasma B cells. Plasma B cells are actively producing antibodies, so their receptors are more likely engaged in responding to an ongoing infection. Interestingly, the abundance of B cell clonal lineages in the bulk and plasma repertoires are strongly correlated (Figure S3A), with Pearson correlations ranging from 0.55–0.88 across individuals and significance $p < 5 \times 10^{-8}$ across individuals; correlations and p values for each individual are shown in Figure S3. The significant correspondence between the bulk and plasma B cell repertoires in Figure S3 indicates that samples from the bulk, which cover a larger depth, are representative of functional immune responses, at least over the course of the infection.

B cell repertoires differ in receptor composition across cohorts

We aimed to investigate whether cohorts with different disease severities can be distinguished by molecular features of their B cell repertoires. Because sequence features of immune receptors are often associated with their binding specificity, we used statistical methods to compare these features at the level of

CellPress
OPEN ACCESS

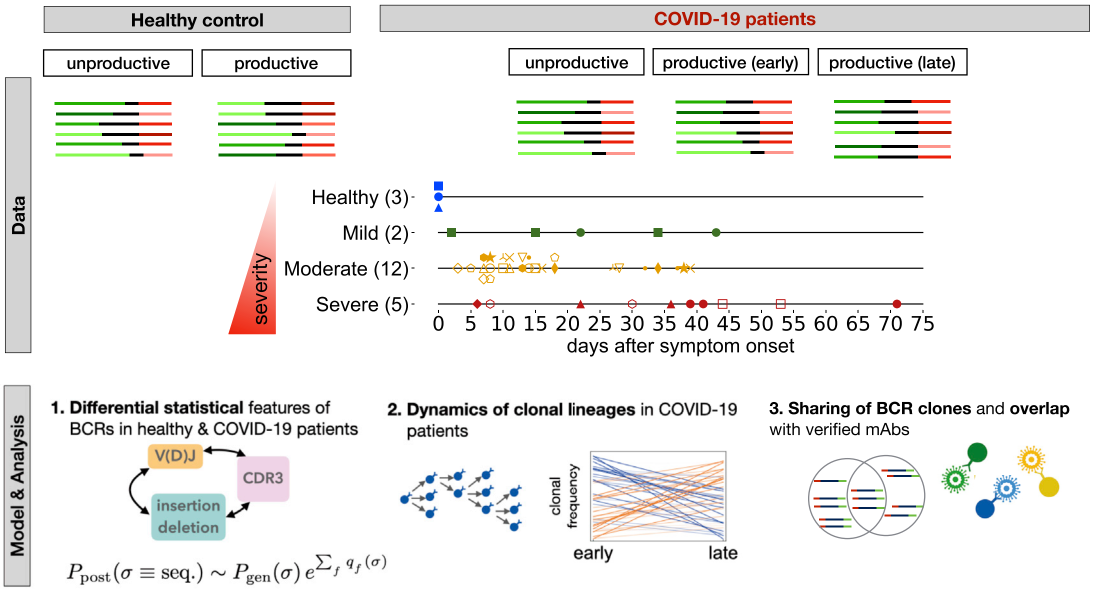

$$P_{\text{post}}(\sigma \equiv \text{seq.}) \sim P_{\text{gen}}(\sigma)\, e^{\sum_f q_f(\sigma)}$$

Figure 1. Roadmap for analysis of BCR repertoires

Top: we collected bulk blood IgG BCR samples from 3 healthy individuals and 2 individuals with mild, 12 with moderate, and 5 with severe symptoms of COVID-19 (different markers and colors). We also collected $CD38^+$ plasma B cells from PBMC samples of 7 individuals in this cohort (6 moderate and 1 severe) and from 7 additional individuals (2 asymptomatic, 3 mild, and 2 moderate) and 3 healthy individuals (Data S1). Samples were collected at different time points during infection (shown in the center for bulk repertoires). We distinguished between productive and unproductive receptors that had frameshifts because of V(D)J recombination. Line segments of varying lengths represent full V(D)J rearrangements (colors). For each individual, we constructed clonal lineages for productive and unproductive BCRs and inferred the naive progenitors of lineages (STAR Methods). Bottom: (1) Using the set of unproductive inferred naive BCRs, we inferred a model to characterize the null probability for generation of receptors $P_{\text{gen}}(\sigma)$(Marcou et al., 2018). We inferred a selection model (Sethna et al., 2020) to characterize the deviation from the null among inferred naive productive BCRs, with the probability of entry to the periphery $P_{\text{post}}(\sigma)$ and selection factors $q_f(\sigma)$ dependent on receptor sequence features. (2) Based on temporal information of sampled BCRs, we identified clonal lineages that expanded significantly during infection. (3) We identified progenitors of clonal lineages shared among individuals and assessed the significance of these sharing statistics based on the probabilities to find each receptor in the periphery. The shared, expanding clonal lineages that contain plasma B cells are likely candidates for secreting responsive antibodies during infection. We verified the reactivity of receptors to SARS-CoV-2 antigenic epitopes using sorted single-cell data. We also identified previously characterized monoclonal antibodies (mAbs) specific to SARS-CoV-2 and SARS-CoV-1.

clonal lineages, including the inferred receptor sequence of lineage progenitors in the bulk (Figures 2 and S2) and in the plasma B cell repertoires (Figure S3) and also the unique sequences in the bulk (Figures S2) and in the plasma B cell repertoires (Figures S3); see Data S1 for details.

Lineage progenitors of IgG repertoires are closest to the ensemble of naive receptors in the periphery. Features of lineage progenitors reflect receptor characteristics that are necessary for activating and forming a clonal lineage in response to an infection. In particular, the subset of lineages that contain plasma BCRs can signal specific responses for antibody production against the infecting pathogen. Statistics of unique sequences in the bulk and the plasma B cell repertoires, on the other hand, contain information about the size of the circulating lineages. Importantly, these statistical ensembles are relatively robust to PCR amplification biases that directly affect read abundances (STAR Methods).

IGHV genes cover a large part of pathogen-engaging regions of BCRs, including the three complementarity-determining regions HCDR1, HCDR2, and a portion of HCDR3. Therefore, we investigated whether there are any differences in V gene usage across cohorts, which may indicate preferences relevant for response to a particular pathogen. We found that the variation in V gene usage among individuals within each cohort was far larger than differences among cohorts in the bulk (Figure 2A) and plasma B cell repertoires (Figure S3B). Data from unique sequences also indicated large background amplitudes because of vast differences in the sizes of lineages within a repertoire (Figures S2A and S3E). Similarly, IGHJ gene usage was also comparable across different cohorts for bulk and plasma B cell repertoires (Figures 2D, S2C, S3D, and S3G). We do not see a significant distinction in statistics of gene usage between the bulk and plasma B cell repertoires (see Figures 2 and S2 for bulk and S3 for plasma B cells). Our results suggest that the SARS-CoV-2 V gene-specific responses are highly individualized at the repertoire level.

HCDR3 is part of the variable chain of BCRs and is often a crucial region in determining specificity. Importantly, HCDR3 is highly variable in its sequence content and length because of insertion and deletion of sequence fragments at the VD and DJ junctions of the germline receptor. Therefore, differential characteristics of the HCDR3 sequence in BCR repertoires of different cohorts can signal preferences for sequence features specific to a class of antigens. We found that HCDR3s of lineages in individuals with COVID-19 with moderate and severe symptoms are significantly longer than in healthy individuals from this study

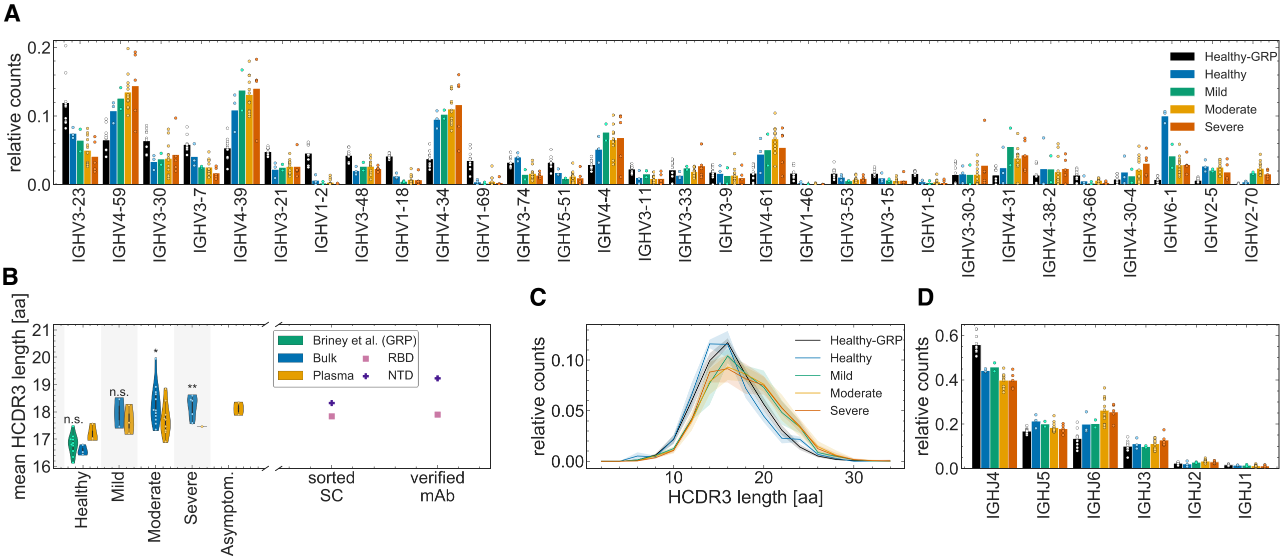

Figure 2. Sequence features of immune receptors in the bulk repertoire across cohorts

(A) The relative counts for IGHV gene usage for inferred naive progenitors of clonal lineages in healthy individuals and those with mild, moderate, and severe COVID-19 symptoms. The bars indicate the use frequency averaged over individuals in each cohort, and dots indicate the variation in V gene frequencies across individuals (biological replicates) within each cohort.

(B and C) Statistics of length of HCDR3 amino acid sequence for different individuals (biological replicates) in each cohort. The violin plots in (B) show the mean HCDR3 length of each individual (dots) in a given cohort (color), with the violin plot cut parameter set to 0.1. The mean HCDR3 lengths of the sorted single cells and verified mAbs (axis) for RBD-reactive (pink squares) and NTD-reactive (purple plus symbols) receptors are shown on the right. Full lines in (C) show distributions averaged over individuals (biological replicates) in each cohort (color), and shading indicates regions containing one standard deviation of variation across individuals within a cohort. One-way ANOVA statistical tests were performed, comparing the mean HCDR3 of all COVID-19 cohorts and the healthy repertoires from the Great Repertoire Project (GRP) dataset (Briney et al., 2019) with the healthy control from this study: healthy-mild: $F_{1,3} = 12.0$, p = 0.04; healthy-moderate: $F_{1,13} = 15.7$, p = 0.0016; healthy-severe: $F_{1,6} = 37.5$, p = 0.00087; healthy-GRP: $F_{1,11} = 0.9$, p = 0.359. Significance cutoffs: n.s. p > 0.01, *p ≤ 0.01, **p < 0.001.

(D) The relative counts for IGHJ gene usage for inferred naive progenitors of clonal lineages in cohorts of healthy individuals and COVID-19 cohorts of individuals with mild, moderate, and severe symptoms. The bars indicate the use frequency averaged over individuals in each cohort, and dots indicate the variation in J gene frequencies across individuals (biological replicates) within each cohort.

See Data S1 for details regarding biological replicates.

and the GRP (Briney et al., 2019; Figures 2B and 2C; one-way ANOVA statistics for differences in mean HCDR3 length: healthy-moderate: $F_{1,13} = 15.7$, $p = 1.6 \times 10^{-3}$; healthy-severe: $F_{1,6} = 37.5$, $p = 8.7 \times 10^{-4}$; GRP-moderate: $F_{1,20} = 34.0$, $p = 1.1 \times 10^{-5}$; GRP-severe: $F_{1,13} = 41.5$, $p = 2.2 \times 10^{-5}$). The difference between HCDR3 length in healthy individuals and individuals with mild symptoms were less significant. These differences are also observed at the level of unique productive BCRs (Figure S2B). These findings are consistent with previous reports of longer HCDR3 lengths in individuals with COVID-19 (Galson et al., 2020; Nielsen et al., 2020; Schultheiß et al., 2020). Despite differences in experimental protocols, the HCDR3 lengths of the healthy cohort from this study and from the GRP (Briney et al., 2019) are comparable with each other (Figures 2B, 2C, and S2B). In addition, we found no significant difference between the HCDR3 length of the unproductive BCR repertoires of healthy individuals and individuals with COVID-19 (Figure S2E), which should reflect biases in generation of receptors prior to functional selection. These findings indicate that BCRs with longer HCDR3s tend to be elicited preferentially in repertoires of individuals responding to SARS-CoV-2. This preference seems to have functional significance because longer HCDR3s are also observed among monoclonal antibodies (mAbs) specific to the RBD and NTD of SARS-CoV-2 (Figure 2B), which were identified in previous studies (Brouwer et al., 2020; Han et al., 2020; Hurlburt et al., 2020; Kreye et al., 2020; Pinto et al., 2020; Robbiani et al., 2020; Wu et al., 2020b; Zost et al., 2020).

Differential selection on B cell repertoires in response to SARS-CoV-2

Longer HCDR3 sequences can introduce more sequence diversity at the repertoire level. Quantifying sequence diversity of a B cell repertoire can be sensitive to the sampling depth in each individual. Despite progress in high-throughput repertoire sequencing techniques, sequenced BCRs still present a highly under-sampled view of the entire repertoire. To characterize the diversity of repertoires and the statistics of sequence features, we inferred models of repertoire generation and selection for entry of receptors into the periphery (STAR Methods; Elhanati et al., 2014; Marcou et al., 2018; Sethna et al., 2020). We first used data from unproductive lineage progenitors of BCRs in the bulk repertoire to infer the highly non-uniform baseline model that characterizes the probability $P_{\text{gen}}(\sigma)$ to generate a given receptor sequence, dependent on its sequence features, including the V, D, and J gene choices and also the inserted and deleted sequences at the VD and DJ junctions (Elhanati et al., 2014;

Marcou et al., 2018; Sethna et al., 2020; Figure 1; STAR Methods). The resulting model reflects the biased preferences in generating BCRs in the bone marrow by V(D)J recombination.

The functional but pathogen-naive BCRs that enter the periphery experience selection through processes known as central tolerance (Janeway et al., 2005). In addition, the inferred progenitors of clonal lineages in the IgG repertoire have undergone antigen-dependent selection that led to expansion of their clonal lineages in response to an infection. These two levels of selection make sequence features of functional lineage progenitors distinct from the pool of unproductive BCRs. In addition, differential selection on receptor features can be used to quantify a distance between repertoires of different cohorts that reflect their functional differences in responses to immune challenges (Isacchini et al., 2021).

To identify these distinguishing sequence features, we inferred a selection model for lineage progenitors (STAR Methods). We characterized the probability to observe a clonal lineage ancestor in the periphery as $P_{\text{post}}(\sigma) \sim P_{\text{gen}}(\sigma) e^{\Sigma_{f:\text{features}}\, q_f(\sigma)}$, which deviates from the inferred generation probability of the receptor $P_{\text{gen}}(\sigma)$ by selection factors $q_f(\sigma)$ (Isacchini et al., 2020a, 2020b, 2021; Sethna et al., 2020). These selection factors $q_f(\sigma)$ depend on sequence features, including IGHV and IGHJ genes, HCDR3 length, and amino acid preferences at different positions in HCDR3 (STAR Methods; Elhanati et al., 2014; Isacchini et al., 2020a, 2020b, 2021; Marcou et al., 2018; Sethna et al., 2020). The inferred selection models are robust to differences in the sample size of the repertoires when enough data are available to train the models (STAR Methods; Figures S4C–S4F). As a result, selection models offer a robust approach to compare functional differences even between repertoires with widely different sample sizes, as is the case for our cohorts (STAR Methods; Figures S4C–S4F).

The distribution of the log probability $\log_{10} P_{\text{post}}(\sigma)$ for the inferred progenitors of clonal lineages observed in individuals from different cohorts is shown in Figure 3A. We find an overabundance of BCR lineages with progenitors that have a low probability of entering the periphery (i.e., a lower $P_{\text{post}}(\sigma)$) in individuals with COVID-19 compared with healthy individuals (Figure 3A). A similar pattern is observed at the level of generation probability $P_{\text{gen}}(\sigma)$ for functional receptors in healthy individuals versus individuals infected with COVID-19 (Figure S4A). Notably, the inferred selection models from the GRP healthy repertoires are comparable with the healthy cohort in this study (Figure S4B). Thus, the overabundance of rare receptors in individuals with COVID-19 is likely to be linked to functional responses associated with stimulation of the repertoires against SARS-CoV-2.

We estimated the diversity of the repertoires in each cohort by evaluating the entropy of receptor sequences generated by the respective repertoire models (STAR Methods). In particular, diverse repertoires that contain B cell lineages with rare receptors (i.e., those with a lower $P_{\text{post}}(\sigma)$), should have larger entropies. We found that immune repertoires are more diverse in individuals with COVID-19 compared with healthy individuals (Figure 3A; STAR Methods). The entropy of BCR bulk repertoires grows with disease severity, from 39.18 bits in the healthy cohort to 40.81 $\pm$0.03 bits in the mild cohort, 41.03 $\pm$0.25 bits in the moderate cohort, and 41.32 $\pm$0.11 bits in the severe cohort (STAR Methods). The error bars show the standard error of the mean obtained by averaging over entropy estimates from different models inferred in each of the COVID-19 cohorts, from repertoires subsampled to the same size as the healthy control (STAR Methods). As indicated in Figure S4, the models inferred from subsampled repertoires are highly consistent within each cohort.

Selection factors $q_f(\sigma)$ determine the deviation in preferences for different sequence features of BCRs in each cohort. A comparison of selection factors among cohorts can characterize their distinctive sequence features. To quantify the selection differences across cohorts, we evaluated the Jensen-Shannon divergence (D_{JS}) between repertoires of different cohorts, which measures the distance between the features of their receptor distributions (Isacchini et al., 2021; STAR Methods). Clustering of the cohorts based on their pairwise D_{JS} indicates that repertoires diverge with growing disease severity and that COVID-19 cohorts are more similar to each other than to the healthy cohort (Figure 3B; STAR Methods).

The inferred selection models enabled us to quantify how different receptor features affect the pairwise D_{JS} of BCR repertoires (STAR Methods). We found that HCDR3 length contributes the most to differences in receptor distributions between healthy and COVID-19 cohorts (Figure 3C), consistent with the significant differences in HCDR3 length distributions shown in Figure 2C. We also found that the amino acid composition of HCDR3 is the second most distinguishing factor between repertoires (Figure 3C), indicating that negatively charged amino acids are slightly suppressed at the center of HCDR3s in COVID-19 cohorts compared with healthy repertoires (Figures 3D–3F). The selection differences of IGHV and IGHJ gene usage between healthy individuals and those with COVID-19 are insignificant (Figures 3C and 3G), consistent with our previous analysis of lineage characteristics in Figures 2A and 2D. HCDR3 length and composition are the molecular features that are most distinguishable at the repertoire level across different cohorts. Nonetheless, further work is necessary to understand the molecular underpinnings that may make these receptor features apt in response to SARS-CoV-2.

Expansion of BCR clonal lineages over time indicates responses to SARS-CoV-2

We examined the dynamics of BCR repertoires in individuals with COVID-19. The binding level (measured by optical density 450 [OD_{450}] in ELISAs) of IgM and IgG antibodies against the RBD or NTD of SARS-CoV-2 increased in most individuals with COVID-19 in our study over the course of their infection (Figures 4A and S5). We expect that the increase in OD_{450} binding level is associated with activation of specific B cells, increasing mRNA production of the corresponding BCRs. Detecting expansion of specific clonal lineages is challenging because of subsampling of the repertoires. Only a limited overlap of BCR lineages was found when we compared data between different time points or between technical replicates of a repertoire sampled at the same time point (Figures S6A and S6B). To identify expanding clonal lineages, we examined lineages only in individuals whose plasma showed an increase in binding level (OD_{450}) to the RBD of SARS-CoV-2 and compared the sequence abundance of those

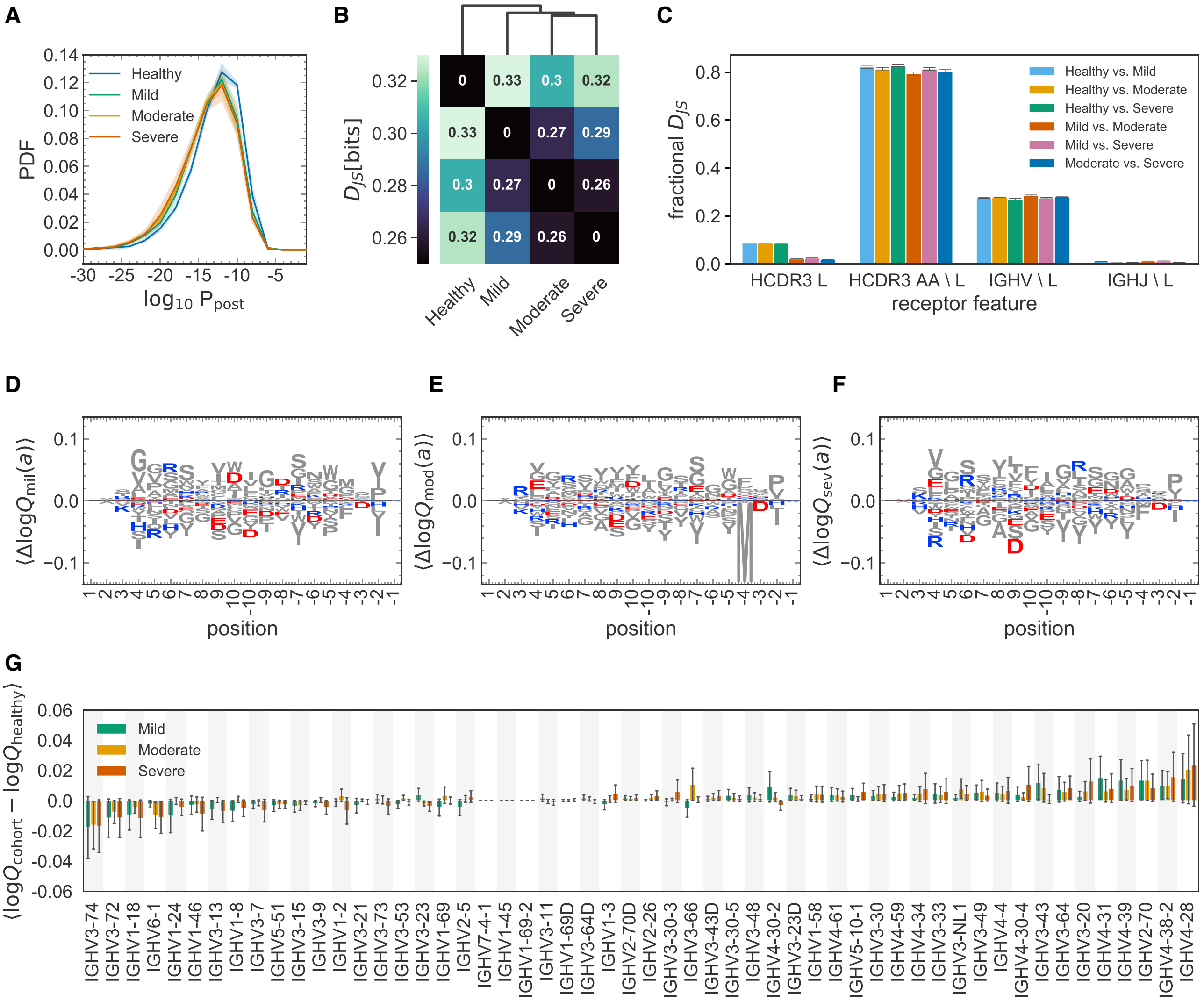

Figure 3. Differential statistics of immune repertoires across cohorts

(A) The distribution of the log probability to observe a sequence σ in the periphery $\log_{10} P_{\mathrm{post}}(\sigma)$ is shown as a normalized probability density function (PDF) for inferred naive progenitors of clonal lineages in cohorts of healthy individuals and the mild, moderate, and severe cohorts of individuals with COVID-19. Full lines show distributions averaged over individuals (biological replicates; Data S1) in each cohort, and shading indicates regions containing one standard deviation of variation among individuals within a cohort.

(B) Clustering of cohorts based on their pairwise Jensen-Shannon divergences (D_{JS}) as a measure of differential selection on cohorts (STAR Methods).

(C) The bar graph shows how incorporating different features into a SONIA selection model contributes to the fractional D_{JS} between models trained on different cohorts. The error bars show the standard deviation of these estimates, using five independent sets of 100,000 generated BCRs for each selection model (STAR Methods).

(D–F) Logo plots show the expected differences in the log-selection factors for amino acid usage, $\langle \Delta \log Q_{\mathrm{cohort}}(a)\rangle = \langle \log Q_{\mathrm{cohort}}(a) - \log Q_{\mathrm{healthy}}(a)\rangle$, for the (D) mild, (E) moderate, and (F) severe COVID-19 cohorts. The expectation values $\langle \cdot \rangle$ are evaluated on the mixture distribution $\frac{1}{2}\left(P_{\mathrm{post}}^{\mathrm{cohort}} + P_{\mathrm{post}}^{\mathrm{healthy}}\right)$. Positively charged amino acids (lysine, K; arginine, R; and histidine, H) are shown in blue, and negatively charged amino acids (aspartate, D, and glutamate, E) are shown in red. All other amino acids are shown in gray. Positions along the HCDR3 are shown up to 10 residues starting from the 3′ (positive values) and 5′ ends (negative values).

(G) The bar graph shows the average mean difference between the log-selection factors for IGHV gene usage for the mild (green), moderate (yellow), and severe (red) COVID-19 cohorts, with the mean differences computed using the mixture distribution $\frac{1}{2}\left(P_{\mathrm{post}}^{\mathrm{cohort}} + P_{\mathrm{post}}^{\mathrm{healthy}}\right)$, and the average is taken over the 30 independently trained SONIA models for each cohort. Error bars show standard deviation of these estimates across the inferred SONIA models (STAR Methods).

lineages in the bulk repertoire that appeared at two or more time points (Figures 4A and S5; STAR Methods). Using a hypothesis test with a false discovery rate of 7.5%, as determined by analyzing technical replicate data (STAR Methods; Figure S6), we detected significant expansion of clonal lineages of receptors harvested from the bulk repertoire within all investigated

CellPress
OPEN ACCESS

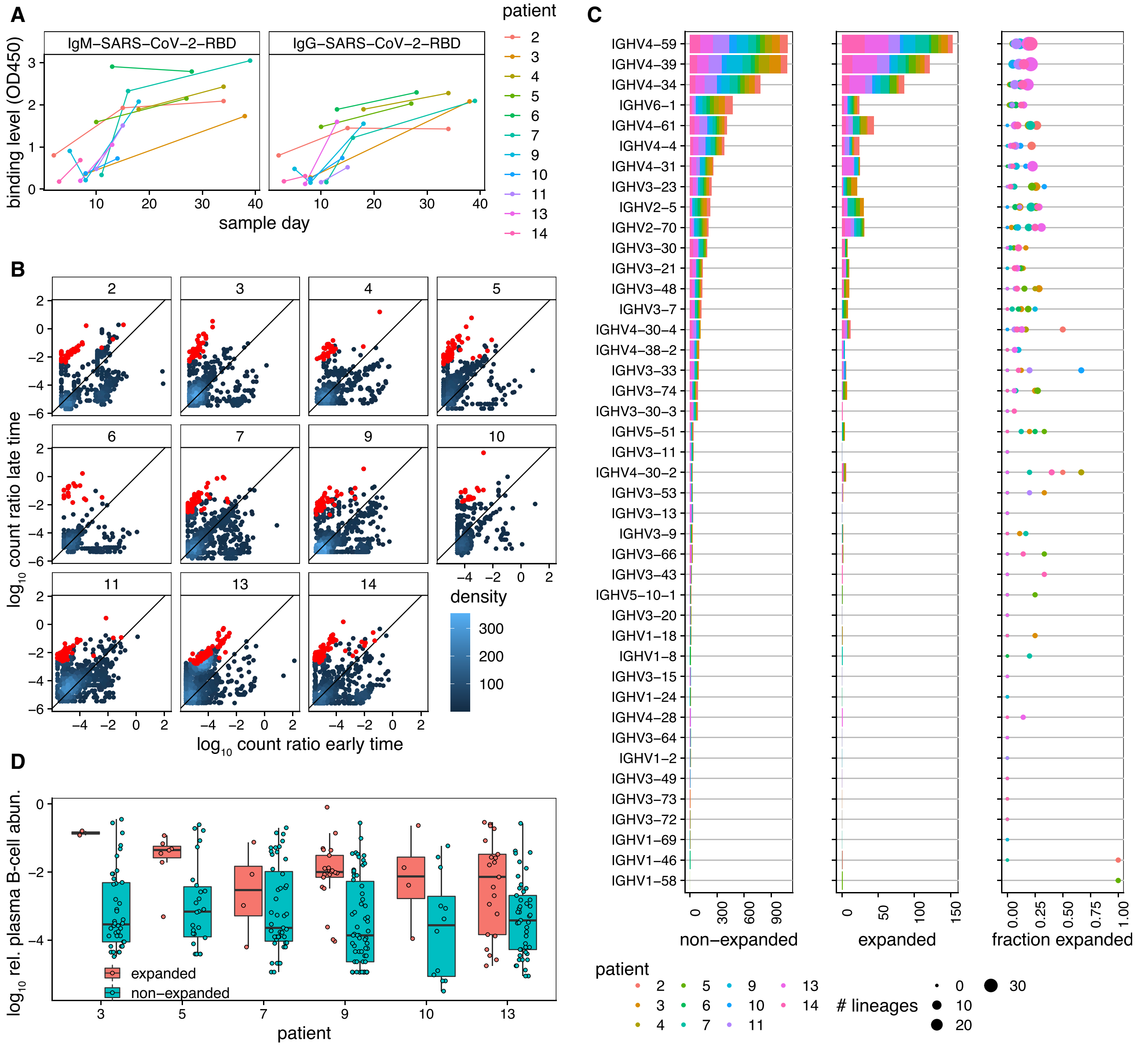

Figure 4. Dynamics of BCR repertoires during infection

(A) The binding level (measured by OD_{450} in ELISA) of the IgM (left) and IgG (right) repertoires to SARS-CoV-2 (RBD) epitopes increases over time in most individuals.

(B) The log ratio of BCR (mRNA) abundance at late time versus early time is shown for all clonal lineages that are present in at least two time points (STAR Methods). Each panel shows dynamics of lineages for a given individual, as indicated in the label. The analysis is shown for individuals for whom the binding level (OD_{450}) of the IgG repertoire increases over time (shown in A). The count density indicates the number of lineages at each point. Lineages that show a significant expansion over time are indicated in red (STAR Methods).

(C) IGHV gene use of lineages is shown for non-expanded (left) and expanded (center) lineages in all individuals (colors). The right panel shows, for each individual (colors), the fraction of expanded lineages with a given IGHV gene as the number of expanded lineages divided by the total number of lineages with that given IGHV gene. The size of the circles indicates the total number of lineages in each category.

(D) Boxplot of $\log_{10}$ relative read abundance in the plasma B cell repertoire (STAR Methods) for expanding (red) and non-expanding (cyan) lineages that contain reads from plasma B cells in different individuals. Receptors from the plasma B cell repertoire are significantly more abundant in expanding lineages in four individuals based on ANOVA test statistics: individual 3: $F_{1,42} = 5.4$, $p = 0.02$; individual 5: $F_{1,31} = 0.5$, $p = 0.5$; individual 7: $F_{1,49} = 0.01$, $p = 0.91$; individual 9: $F_{1,42} = 4.1$, $p = 0.04$; individual 10: $F_{1,42} = 2.9$, $p = 0.1$; individual 13: $F_{1,64} = 7.7$, $p = 0.007$.

individuals. The results reflect a dynamic repertoire in all individuals, ranging from 5%–15% of lineages with significantly large changes in sequence abundances over time (Figures 4 and S6). The expanding lineages have an HCDR3 length comparable with the rest of the repertoire in individuals with COVID-19 (Figure S6). Moreover, the expanding lineages show V gene

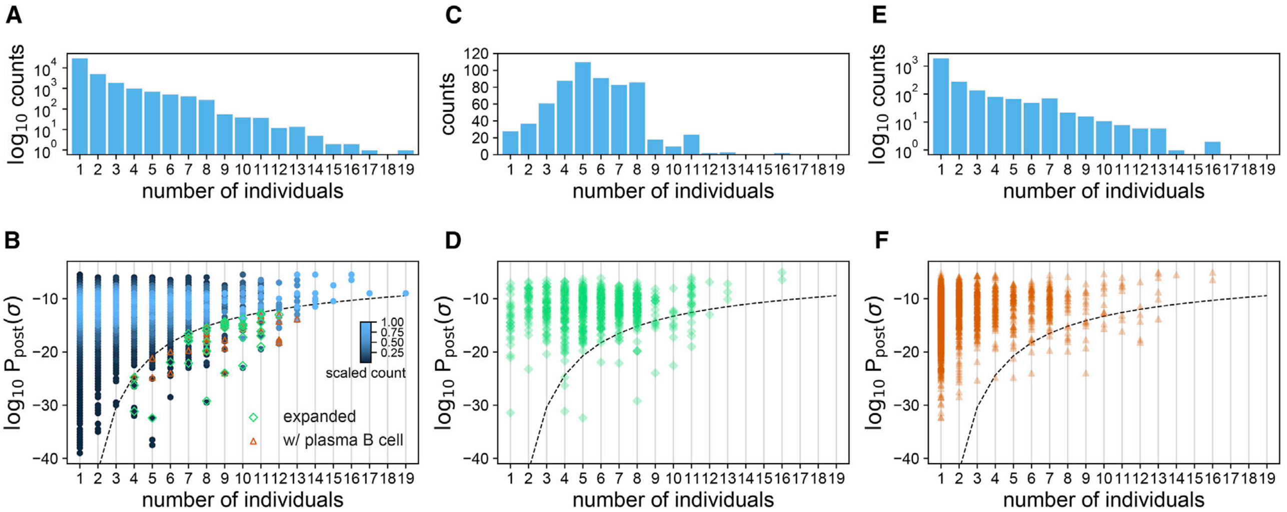

Figure 5. Sharing of BCRs among individuals

(A) The histogram shows the number of clonal lineages that share a common progenitor in a given number of individuals, indicated on the horizontal axis.

(B) The density plot shows the distribution of $\log_{10} P_{post}$ for progenitors of clonal lineages shared in a given number of individuals, indicated on the horizontal axis. The histogram bin size is 0.5. The scaling of sequence counts sets the maximum of the density in each column to one. Sharing of rare lineages with $\log_{10} P_{post}$ below the dashed line is statistically significant (STAR Methods). Green diamonds indicate clonal lineages below the dashed line with significant expansion in at least one of the individuals. Orange triangles indicate clonal lineages below the dashed line that contain reads from the plasma B cell repertoire in at least one of the individuals.

(C and E) Histograms showing the numbers of clonal lineages that share a common progenitor in a given number of individuals that have expanded significantly during infection in at least one of the individuals (C) or contained reads from the plasma B cell repertoire in at least one of the individuals (E).

(D and F) Scatterplots with transparent overlapping markers show $\log_{10} P_{post}$ for progenitors of clonal lineages shared in a given number of individuals that have expanded (D) or contain reads from the plasma B cell repertoire (F) in at least one individual. The dashed line is similar to (B).

preferences comparable with previously identified antibodies against SARS-CoV-2 (RBD). This includes the abundance of IGHV4-59, IGHV4-39, IGHV3-23, IGHV3-53, IGH3-66, IGHV2-5, and IGHV2-70 (Brouwer et al., 2020; Ju et al., 2020; Pinto et al., 2020; Rogers et al., 2020). However, these preferences in V gene usage among expanding lineages are also comparable with the overall biases in V gene usage within individuals, and expanded lineages roughly make up 25% of lineages with a given V gene (Figure 4C). Therefore, our results suggest that the overall response to SARS-CoV-2 is not driven by only specific classes of IGHV genes.

We expect clonal expansions to reflect responses to SARS-CoV-2 during infection. Indeed, we observe that expanding lineages (based on the bulk data) show an over-representation of receptors harvested from plasma B cells, which are likely to be associated with antibody-secreting B cells (Figure 4D; STAR Methods); specific p values for each individual are reported in the legend of Figure 4D.

Sharing of BCRs among individuals

Despite the vast diversity of BCRs, we observe a substantial number of identical progenitors of BCR clonal lineages among individuals with COVID-19 (Figure 5) and among healthy individuals from our dataset and from the GRP (Figure S7). Previous work has also identified sharing of BCRs among individuals with COVID-19, which was interpreted by the authors as evidence of large-scale convergence of immune responses (Galson et al., 2020; Nielsen et al., 2020; Schultheiß et al., 2020). Although BCR sharing can be due to convergent responses to common antigens, it can also arise from convergent recombination leading to the same receptor sequence (Elhanati et al., 2018; Pogorelyy et al., 2018a) or from experimental biases. Therefore, it is imperative to formulate a statistical model to quantify the significance of BCR sharing. Convergent recombination defines a null expectation for the amount of sharing within a cohort based on only the underlying biases for receptor generation within a repertoire (Elhanati et al., 2018; Pogorelyy et al., 2018a; STAR Methods). Intuitively, sharing is more likely among commonly generated receptors (i.e., with a high $P_{post}(\sigma)$) and within cohorts with larger sampling (STAR Methods). Consequently, rare receptors (i.e., with a low $P_{post}(\sigma)$) that are shared among individuals in a common disease group can signal commonality in function and a response to a common antigen, as observed previously for T cell receptors (TCRs) in response to a yellow fever vaccine (Pogorelyy et al., 2018b), cytomegalovirus (CMV), and diabetes (Pogorelyy et al., 2018a).

We used the receptors' probabilities $P_{post}(\sigma)$ to assess the significance of sharing by identifying a probabilistic threshold to limit the shared outliers among individuals with COVID-19 (dashed line in Figure 5) and healthy individuals (dashed lines in Figure S7). Of a total of 40,128 (unique) progenitors of clonal lineages reconstructed from the pooled bulk+plasma B cell repertoires (Figure 5A; Data S1), we found 10,146 progenitors to be shared among at least two individuals, and 761 of these lineages contained receptors found in plasma B cells of at least one

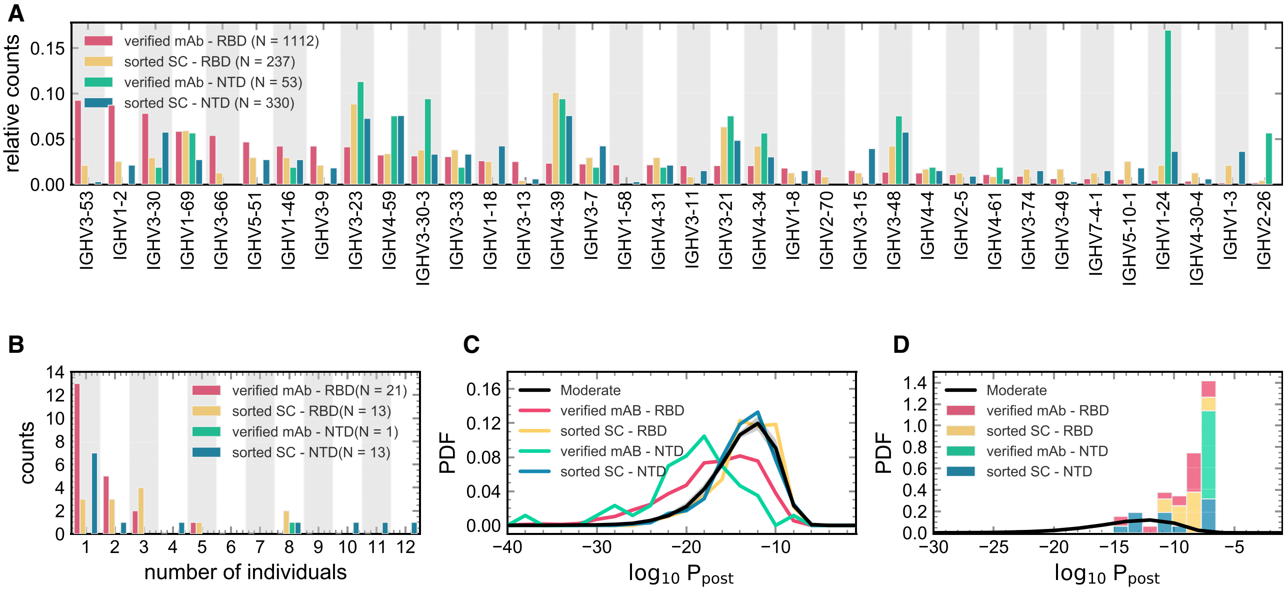

Figure 6. Statistics of BCRs reactive to RBD and NTD epitopes

(A) Relative counts for IGHV gene usage for known mAbs (Data S3) reactive to RBD (pink) and NTD (green) epitopes of SARS-CoV-2 and for receptors obtained from single-cell sequencing of the pooled sample from all individuals (STAR Methods), sorted for RBD (yellow) and NTD (blue) epitopes.

(B) The histogram shows the number of NTD-sorted receptors from single cell sequencing (Data S2) and RBD- and NTD-specific verified mAbs (Data S3) found in the bulk+plasma B cell repertoires of a given number of individuals (STAR Methods), indicated on the horizontal axis.

(C) The distribution of the log probability to observe a sequence σ in the periphery $\log_{10} P_{post}(\sigma)$ is shown as a normalized PDF for inferred naive progenitors of known RBD- and NTD-specific mAbs and for RBD- and NTD-sorted receptors from single-cell sequencing. $P_{post}(\sigma)$ values were evaluated based on the repertoire model created from individuals with moderate symptoms. The corresponding $\log_{10} P_{post}$ distribution for bulk repertoires of the moderate cohort (similar to Figure 3A) is shown in black as a reference.

(D) Similar to (C) but restricted to receptors that are found in the bulk+plasma repertoire of at least one individual in the cohort (Data S2 and S3).

Colors are consistent between panels, and the number of samples used in each panel is indicated in the legend.

individual. 167 of the 10,146 lineage progenitors were classified as rare, having a probability of occurrence below the indicated threshold (dashed line in Figure 5B). 30 of these contain receptors harvested from plasma B cells, indicating a significant over-abundance of plasma B cells among the rare, shared receptors ($p = 7.2 \times 10^{-6}$) (Figures 5E and 5F). Moreover, we found that 615 lineages shared a common sequence ancestor in at least two individuals and had expanded in at least one of the individuals (Figures 5C and 5D). 38 of these shared, expanding lineages stemmed from rare naive progenitors (below the dashed line in Figures 5B and 5D), eight of which contain receptors found in plasma B cells of at least one individual. The over-abundance of plasma BCRs in the rare shared, expanding lineages is significant ($p = 0.04$). The sharing of these rare, expanding BCRs among individuals with COVID-19, with an over-abundance of receptors associated with antibody production in the plasma B cell data, indicates a potentially convergent response to SARS-CoV-2; these receptors are listed in Data S2.

We found that 24% of receptors in the 38 rare shared, expanding lineages contain multiple cysteines in their HCDR3s, in contrast to only 10% of the receptors in the whole repertoire. Sequence patterns with cysteine pairs in the HCDR3 have been associated with stabilization of the HCDR3 loop by forming disulfide bonds with particular patterns and spacings of the cysteines (Lee et al., 2014; Prabakaran and Chowdhury, 2020). Disulfide bonds in the HCDR3 can decrease the conformational flexibility of the loop, thus decreasing the entropic cost of binding to improve the affinity of the receptor (Almagro et al., 2012). The significantly larger fraction of multi-cysteine HCDR3s among the candidate SARS-CoV-2-responsive receptors ($p = 0.013$ based on binomial sampling) indicates an underlying molecular mechanism for developing a potent response to SARS-CoV-2.

Presence of SARS-CoV-2- and SARS-CoV-1-specific neutralizing antibodies within repertoires

To further investigate the functional response in the repertoire of individuals with COVID-19, we performed single-cell sequencing on pooled samples from all individuals, sorted for reactivity to RBD or NTD epitopes of SARS-CoV-2 (STAR Methods). This analysis suggests that about 0.2% of these single cells are RBD reactive as opposed to only 0.02% that are NTD reactive (Figure S1). This inferred fraction of reactive antibodies is consistent with previous estimates (Kreer et al., 2020a).

We characterized the sequence features of RBD- and NTD-sorted antibodies. The IGHV gene usage of these reactive receptors is shown in Figure 6 and is compared with gene usage in mAbs identified in previous studies (Brouwer et al., 2020; Han et al., 2020; Hurlburt et al., 2020; Kreye et al., 2020; Pinto et al., 2020; Robbiani et al., 2020; Wu et al., 2020b; Zost et al., 2020). Despite the broad range of IGHV gene usage associated with epitope reactivity, sorted single cells show IGHV gene preferences common to the previously identified mAbs against

SARS-CoV-2 epitopes. This includes an abundance of IGHV1-69, IGHV4-59, IGHV3-30-3, IGHV3-33, IGHV1-18, IGHV5-51, and IGHV1-46 against the RBD and IGHV3-23, IGHV4-59, IGHV4-39, IGHV3-21, and IGHV3-48 against the NTD (Figure 6A). Similarly, we observe consistent biases in V and J gene usage of the κ and λ light chains for the sorted single cells and verified mAbs (Figure S8). Moreover, the HCDR3 length distributions of the sorted single cells are comparable to those of the verified mAbs (Figure S8). The average lengths of the HCDR3 for the verified mAbs and sorted single-cell receptors are comparable to those of bulk repertoires from individuals with COVID-19, which are significantly longer than those of healthy individuals (Figure 2B).

To characterize how SARS-CoV-2-reactive receptors make up individuals' repertoires, we mapped the heavy chain receptors from the sorted single cells onto BCR lineages constructed from the bulk+plasma B cell data in individuals with COVID-19 (STAR Methods; Data S2). We found that 13 (of 237) RBD-sorted and 13 (of 330) NTD-sorted antibodies from the single cells matched receptor lineages in at least one individual (Figure 6B). Interestingly, we found broad sharing of these antibodies with 10 RBD- and 6 NTD-sorted single cells present in at least two individuals (Figure 6B).

In repertoires of individuals with COVID-19, we found that several HCDR3s matched with SARS-CoV-2-specific mAbs that have been isolated previously in other studies (Brouwer et al., 2020; Han et al., 2020; Hurlburt et al., 2020; Kreye et al., 2020; Pinto et al., 2020; Robbiani et al., 2020; Wu et al., 2020b; Zost et al., 2020). Specifically, a total of 20 mAb families specific to SARS-CoV-2 epitopes were found to be close in sequence to HCDR3s in our data (with up to one amino acid difference), among which are 14 RBD-specific, one NTD-specific, and five S1-specific (reactive to the RBD or NTD) mAbs (Figure 6B; Data S3). Interestingly, nine of these mAbs are shared among at least two individuals, and the NTD-specific antibody is found in eight individuals (Figure6B).

We also found that two individuals with COVID-19 had exact HCDR3 matches to a previously identified antibody, S304, that has cross-reactivity to SARS-CoV-1 and SARS-CoV-2 (Pinto et al., 2020). We observed in one patient an HCDR3 with only one amino acid difference to this antibody (Data S3). Importantly, the plasma in these individuals showed a substantial binding level (OD_{450}) to SARS-CoV-1 (Figure S5), which indicates possible cross-reactive antibody responses to SARS-CoV-1 and SARS-CoV-2.

We investigated the matches between the RBD- and NTD-sorted single-cell receptors with verified mAbs from previous studies (Brouwer et al., 2020; Han et al., 2020; Hurlburt et al., 2020; Kreye et al., 2020; Pinto et al., 2020; Robbiani et al., 2020; Wu et al., 2020b; Zost et al., 2020). Although we found no matches between the heavy chain CDR3 of sorted single-cell receptors and the verified mAbs, we found a large number of matches between the κ and λ light chain CDR3s of the sets (Figure S8). Notably, 59 of 142 IG_κ and 47 of 110 IG_λ from the RBD-reactive single cells and 1 of 202 IG_κ and 22 of 155 IG_λ from the NTD-reactive single cells matched to light chain CDR3s of mAbs in those respective subsets (Figure S8). Given the low sequence diversity of light chain receptors, it remains to be seen whether these matches between the light chain mAbs and sorted single cells are statistically significant—a question that requires modeling the generation and selection of the light chain receptor repertoires.

Last, we observed that previously verified mAbs have a lower probability $P_{\text{post}}(\sigma)$ of generation and entry to the periphery compared with the overall repertoire (Figure 6C). This is partly expected because the selection models used to evaluate these probabilities were trained on different repertoires than those from which these mAbs were originally harvested. Consistently, the evaluated probabilities for the sorted single-cell receptors are within the range for the bulk repertoire (Figure 6C) because the two datasets were derived from the same cohort. Notably, all the verified mAbs and the sorted single-cell receptors that we can match to the individuals' repertoires have a relatively high probability $P_{\text{post}}(\sigma)$ (Figure 6D). This is not surprising as it is unlikely to observe rare BCRs to be shared across different cohorts. Overall, our results are encouraging for vaccine development because they indicate that even common antibodies can confer responses specific to SARS-CoV-2.

DISCUSSION

COVID-19 will remain an ongoing threat to public health until an effective SARS-CoV-2 vaccine is available globally. Understanding the human B cell immune response to SARS-CoV-2 is critical for vaccine development and assessment (Wec et al., 2020a). A repertoire of immune receptor sequences represents a unique snapshot of the history of immune responses in an individual (Boyd et al., 2009; Georgiou et al., 2014; Kreer et al., 2020b; Robins, 2013), and changes in a repertoire during an infection can signal responses specific to pathogens (Horns et al., 2019; Nourmohammad et al., 2019). Identifying signatures of a functional response to a given pathogen from a pool of mostly unspecific BCRs collected from the blood is challenging—it is like finding a needle in a haystack. Therefore, principled statistical inference approaches are necessary to extract functional signals from such data. Here we systematically characterize the B cell repertoire response to SARS-CoV-2 in individuals with different severities of COVID-19 by combining evidence from the overall statistics of repertoires with dynamics of clonal lineages during infection and sharing of immune receptors among individuals.

At the repertoire level, we showed that the HCDR3 of BCRs in individuals with COVID-19 are significantly longer than the HCDR3 in healthy individuals and that the amino acid composition of this receptor region varies among cohorts of individuals with mild, moderate, and severe symptoms. We observed large-scale sharing of BCRs among individuals with COVID-19, consistent with previous findings in those with COVID-19 (Galson et al., 2020; Nielsen et al., 2020; Schultheiß et al., 2020). Sharing of BCRs among individuals can signal common immune responses to a pathogen. However, BCR sharing can also be due to convergent recombination leading to the same receptor sequence or other experimental biases that influence statistics of shared sequences. These statistical nuances can substantially sway conclusions drawn from the sharing analysis and should be carefully accounted for. Here we established a null

expectation of BCR sharing due to convergent recombination by inferring a model of receptor generation and selection. Our analysis identified a subset of rare BCRs shared among individuals with COVID-19, which appears to signal convergent responses to SARS-CoV-2.

Bulk B cell repertoires predominantly contain a mixture of naive, memory, and plasma B cells. At the early stages of viral infection, antigen-specific plasma B cells may develop that act as antibody factories and confer neutralization against the infecting pathogen (Wrammert et al., 2008). Almost all prior work on immune repertoires has focused on bulk repertoires, which are often easier to sample and analyze. Moreover, functional studies, using single-cell sequencing of antigen-sorted BCRs, have often been disconnected from large-scale analysis of receptor repertoires. Our study synergizes data from bulk and plasma B cell sequencing with antigen-sorted single-cell BCRs to draw a more complete picture of the human immune response to SARS-CoV-2. Importantly, our joint longitudinal analysis of the bulk and plasma B cell repertoires in individuals with COVID-19 provides insight into the dynamics of antigen-specific B cells as well as the statistics of receptor sequence features associated with responses to SARS-CoV-2.

In addition to the statistics of repertoires, we observed that the activity of many B cell lineages (i.e., mRNA production) in individuals with COVID-19 increases during infection, accompanied by an increase in the binding level (OD_{450}) of the individuals' plasma to the RBD and NTD of SARS-CoV-2. The dynamics of clonal lineages during an infection provide significant insights into the characteristics of responsive antibodies (Horns et al., 2019; Nourmohammad et al., 2019). By taking advantage of data collected at multiple time points in most individuals, we identified expanded lineages shared among individuals and found 38 clonal lineages that are candidates for a response specific to SARS-CoV-2 antigens (Figure 5; Data S2). Importantly, the over-representation of plasma B cells among these shared expanding lineages signifies their potential role in mounting protective antibody responses against SARS-CoV-2. It should be noted that none of these 38 clonal lineages matched with the verified mAbs. This is in part expected because the verified mAbs that matched the bulk repertoires have relatively high probabilities P_{post} (Figure 6D), whereas these 38 lineages are chosen explicitly to be rare.

Our analysis of repertoire dynamics has identified a large-scale expansion of B cell clonal lineages (5%−15% of lineages) over the course of COVID-19 infection. However, it is hard to imagine that all of these expanding clones that account for a sizeable portion of the repertoire are engaged in responding specifically to SARS-CoV-2. In contrast, our single-cell analysis identified about 0.2% of receptors as reactive to RBD and 0.02% as reactive to NTD epitopes (Figure S1)—an estimate that is consistent with previous findings (Kreer et al., 2020a). This disparity raises an open question: why do we observe such a large-scale expansion of clonal lineages during an acute immune response?

Identifying antibodies with cross-reactive neutralization abilities against viruses in the SARS family is of significant interest. Although cross-neutralization antibodies have been isolated from individuals with COVID-19 (Brouwer et al., 2020; Liu et al., 2020a; Zhou et al., 2020), it remains unclear how prevalent they are. In nine individuals, we see a substantial increase in the binding level (OD_{450}) of their plasma to SARS-CoV-1 epitopes over the course of COVID-19 infection. In three individuals, we identify a BCR identical to the heavy chain of antibody S304 (Pinto et al., 2020), which has been isolated previously from an individual who recovered from a SARS-CoV-1 infection. This antibody has been shown to be moderately cross-reactive to SARS-CoV-1 and SARS-CoV-2, and our results indicate a possibility for such cross-reactive antibodies to emerge naturally in response to SARS-CoV-2 (Brouwer et al., 2020; Lv et al., 2020; Rogers et al., 2020). Our findings provide substantial insight into and strong implications for devising vaccines and therapies with a broad applicability against SARS-CoV-2.

STAR★METHODS

Detailed methods are provided in the online version of this paper and include the following:

- KEY RESOURCES TABLE
- RESOURCE AVAILABILITY
  - Lead contact
  - Materials availability
  - Data and code availability
- EXPERIMENTAL MODEL AND SUBJECT DETAILS
  - Cell lines
  - Patients and samples
- METHOD DETAILS
  - PBMC isolation
  - RNA extraction and reverse transcription
  - Amplification of B cell repertoire from the samples by PCR
  - Protein expression and purification
  - $CD38^+$ plasma B cell enrichment
  - RBD and NTD protein specific binding B cell enrichment
  - Single B cell 5′ mRNA and VDJ sequencing
  - ELISA
  - BCR preprocessing
  - BCR error correction
  - BCR annotation
  - Unproductive BCRs
  - Clonal lineage reconstruction
  - Mapping of single-cell data onto reconstructed clonal lineages
  - Inference of generation probability and selection for BCRs
  - Characterizing the robustness of selection inference
  - Characterizing repertoire diversity
  - Comparing selection between repertoires of cohorts
  - Clonal lineage expansion
  - Significance of BCR sharing among individuals
- QUANTIFICATION AND STATISTICAL ANALYSIS

SUPPLEMENTAL INFORMATION

Supplemental information can be found online at https://doi.org/10.1016/j.celrep.2021.109173.

ACKNOWLEDGMENTS

We are grateful for discussions with Maria Ruiz Ortega on selection inference in BCR repertoires. This work was supported by Deutsche Forschungsgemeinschaft grant (SFB1310) on Predictability in Evolution (to A.N., Z.M., J.O., and G.I.); MPRG funding from the Max Planck Society (to A.N., Z.M., J.O., and G.I.); the Royalty Research Fund at the University of Washington (to A.N. and Z.M.); NIH NIAID F31AI150163 (to W.S.D.); a Calmette and Yersin scholarship from the Pasteur International Network Association (to H.L.); Bill and Melinda Gates Foundation OPP1170236 (to I.A.W.); a startup fund at the University of Illinois at Urbana-Champaign (to N.C.W.); the National Institutes of Health (contract HHSN272201400006C to J.S.M.P.); the National Natural Science Foundation of China (NSFC)/Research Grants Council (RGC) Joint Research Scheme (N_HKU737/18 to C.K.P.M. and J.S.M.P.); and the Research Grants Council of the Hong Kong Special Administrative Region, China (project T11-712/19-N to J.S.M.P.). We acknowledge support from the clinicians who facilitated this study, including Drs. Wai Shing Leung, Jacky Man Chun Chan, Thomas Shiu Hong Chik, Chris Yau Chung Choi, John Yu Hong Chan, Daphne Pui-Lin Lau, and Ying Man Ho; the dedicated clinical team at the Infectious Diseases Centre, Princess Margaret Hospital, Hospital Authority of Hong Kong; and the individuals who kindly consented to participate in this investigation. We also thank the Center for PanorOmic Sciences (CPOS), LKS Faculty of Medicine, and University of Hong Kong for support with next-generation sequencing and acknowledge use of the computational infrastructure provided by the Hyak supercomputer system funded by the Student Technology Fund (STF) at the University of Washington.

AUTHOR CONTRIBUTIONS

Z.M., H. Lv, J.O., I.A.W., J.S.M.P., N.C.W., A.N., and C.K.P.M. conceived and designed the study. O.T.-Y.T. organized recruitment, data collection, and sampling. H. Lv, G.K.Y., W.W.N., and C.K.P.M. prepared the next-generation sequencing libraries and performed the ELISA experiments. M.Y., H. Liu, and N.C.W. expressed and purified the proteins. Z.M., J.O., W.S.D., G.I., and A.N. analyzed the data and performed the modeling work and statistical inference. Z.M., H. Lv, N.C.W., A.N., and C.K.P.M. wrote the paper. All authors reviewed and edited the paper.

DECLARATION OF INTERESTS

The authors declare no competing interests.

Received: December 4, 2020
Revised: April 5, 2021
Accepted: May 4, 2021
Published: May 7, 2021

REFERENCES

Almagro, J.C., Raghunathan, G., Beil, E., Janecki, D.J., Chen, Q., Dinh, T., LaCombe, A., Connor, J., Ware, M., Kim, P.H., et al. (2012). Characterization of a high-affinity human antibody with a disulfide bridge in the third complementarity-determining region of the heavy chain. J. Mol. Recognit. *25*, 125–135.

Barnes, C.O., West, A.P., Jr., Huey-Tubman, K.E., Hoffmann, M.A.G., Sharaf, N.G., Hoffman, P.R., Koranda, N., Gristick, H.B., Gaebler, C., Muecksch, F., et al. (2020). Structures of human antibodies bound to SARS-CoV-2 spike reveal common epitopes and recurrent features of antibodies. Cell *182*, 828–842.e16.

Boyd, S.D., Marshall, E.L., Merker, J.D., Maniar, J.M., Zhang, L.N., Sahaf, B., Jones, C.D., Simen, B.B., Hanczaruk, B., Nguyen, K.D., et al. (2009). Measurement and clinical monitoring of human lymphocyte clonality by massively parallel VDJ pyrosequencing. Sci. Transl. Med. *1*, 12ra23.

Briney, B., and Burton, D.R. (2018). Massively scalable genetic analysis of antibody repertoires. bioRxiv. https://doi.org/10.1101/447813.

Briney, B., Inderbitzin, A., Joyce, C., and Burton, D.R. (2019). Commonality despite exceptional diversity in the baseline human antibody repertoire. Nature *566*, 393–397.

Brouwer, P.J.M., Caniels, T.G., van der Straten, K., Snitselaar, J.L., Aldon, Y., Bangaru, S., Torres, J.L., Okba, N.M.A., Claireaux, M., Kerster, G., et al. (2020). Potent neutralizing antibodies from COVID-19 patients define multiple targets of vulnerability. Science *369*, 643–650.

Burnet, F.M. (1959). The clonal selection theory of acquired immunity (Vanderbilt University Press).

Burnet, F.M. (1960). Immunity as an aspect of general biology. In Mechanisms of Antibody Formation, M. Holub and J. Jaroskova, eds. (Academia Publishing House of the Czech Academy of Sciences), pp. 15–21.

Cao, Y., Su, B., Guo, X., Sun, W., Deng, Y., Bao, L., Zhu, Q., Zhang, X., Zheng, Y., Geng, C., et al. (2020). Potent neutralizing antibodies against SARS-CoV-2 identified by high-throughput single-cell sequencing of convalescent patients' B cells. Cell *182*, 73–84.e16.

Chi, X., Yan, R., Zhang, J., Zhang, G., Zhang, Y., Hao, M., Zhang, Z., Fan, P., Dong, Y., Yang, Y., et al. (2020). A neutralizing human antibody binds to the N-terminal domain of the spike protein of SARS-CoV-2. Science *369*, 650–655.

Cyster, J.G., and Allen, C.D.C. (2019). B cell responses: cell interaction dynamics and decisions. Cell *177*, 524–540.

DeWitt, W.S., Emerson, R.O., Lindau, P., Vignali, M., Snyder, T.M., Desmarais, C., Sanders, C., Utsugi, H., Warren, E.H., McElrath, J., et al. (2015). Dynamics of the cytotoxic T cell response to a model of acute viral infection. J. Virol. *89*, 4517–4526.

Elhanati, Y., Murugan, A., Callan, C.G., Jr., Mora, T., and Walczak, A.M. (2014). Quantifying selection in immune receptor repertoires. Proc. Natl. Acad. Sci. USA *111*, 9875–9880.

Elhanati, Y., Sethna, Z., Callan, C.G., Jr., Mora, T., and Walczak, A.M. (2018). Predicting the spectrum of TCR repertoire sharing with a data-driven model of recombination. Immunol. Rev. *284*, 167–179.

Ellinghaus, D., Degenhardt, F., Bujanda, L., Buti, M., Albillos, A., Invernizzi, P., Fernández, J., Prati, D., Baselli, G., Asselta, R., et al.; Severe Covid-19 GWAS Group (2020). Genomewide association study of severe COVID-19 with respiratory failure. N. Engl. J. Med. *383*, 1522–1534.

Galson, J.D., Schaetzle, S., Bashford-Rogers, R.J.M., Raybould, M.I.J., Kovaltsuk, A., Kilpatrick, G.J., Minter, R., Finch, D.K., Dias, J., James, L., et al. (2020). Deep sequencing of B cell receptor repertoires from COVID-19 patients reveals strong convergent immune signatures. bioRxiv. https://doi.org/10.1101/2020.05.20.106294.

Georgiou, G., Ippolito, G.C., Beausang, J., Busse, C.E., Wardemann, H., and Quake, S.R. (2014). The promise and challenge of high-throughput sequencing of the antibody repertoire. Nat. Biotechnol. *32*, 158–168.

Guan, W.J., Ni, Z.Y., Hu, Y., Liang, W.H., Ou, C.Q., He, J.X., Liu, L., Shan, H., Lei, C.L., Hui, D.S.C., et al.; China Medical Treatment Expert Group for Covid-19 (2020). Clinical characteristics of coronavirus disease 2019 in China. N. Engl. J. Med. *382*, 1708–1720.

Gupta, N.T., Adams, K.D., Briggs, A.W., Timberlake, S.C., Vigneault, F., and Kleinstein, S.H. (2017). Hierarchical clustering can identify B Cell clones with high confidence in Ig repertoire sequencing data. J. Immunol. *198*, 2489–2499.

Hachim, A., Kavian, N., Cohen, C.A., Chin, A.W., Chu, D.K., Mok, C.K.P., Tsang, O.T., Yeung, Y.C., Perera, R.A., Poon, L.L., et al. (2020). Beyond the spike: identification of viral targets of the antibody response to SARS-CoV-2 in COVID-19 patients. medRxiv. https://doi.org/10.1101/2020.04.30.20085670.

Han, X., Wang, Y., Li, S., Hu, C., Li, T., Gu, C., Wang, K., Shen, M., Wang, J., Hu, J., et al. (2020). A rapid and efficient screening system for neutralizing antibodies and its application for the discovery of potent neutralizing antibodies to SARS-CoV-2 S-RBD. bioRxiv. https://doi.org/10.1101/2020.08.19.253369.

Hansen, J., Baum, A., Pascal, K.E., Russo, V., Giordano, S., Wloga, E., Fulton, B.O., Yan, Y., Koon, K., Patel, K., et al. (2020). Studies in humanized mice and convalescent humans yield a SARS-CoV-2 antibody cocktail. Science *369*, 1010–1014.

Horns, F., Vollmers, C., Dekker, C.L., and Quake, S.R. (2019). Signatures of selection in the human antibody repertoire: Selective sweeps, competing subclones, and neutral drift. Proc. Natl. Acad. Sci. USA *116*, 1261–1266.

Hurlburt, N.K., Seydoux, E., Wan, Y.-H., Edara, V.V., Stuart, A.B., Feng, J., Suthar, M.S., McGuire, A.T., Stamatatos, L., and Pancera, M. (2020). Structural basis for potent neutralization of SARS-CoV-2 and role of antibody affinity maturation. Nat. Commun. *11*, 5413.

Isacchini, G., Sethna, Z., Elhanati, Y., Nourmohammad, A., Walczak, A.M., and Mora, T. (2020a). Generative models of T-cell receptor sequences. Phys. Rev. E *101*, 062414.

Isacchini, G., Olivares, C., Nourmohammad, A., Walczak, A.M., and Mora, T. (2020b). SOS: online probability estimation and generation of T-and B-cell receptors. Bioinformatics *36*, 4510–4512.

Isacchini, G., Walczak, A.M., Mora, T., and Nourmohammad, A. (2021). Deep generative selection models of T and B cell receptor repertoires with soNNia. Proc. Natl. Acad. Sci. USA *118*, e2023141118.

Janeway, C.A., Travers, P., Walport, M., and Shlomchik, M.J. (2005). Immunobiology: the immune system in health and disease, Sixth Edition (Garland Science).

Ju, B., Zhang, Q., Ge, J., Wang, R., Sun, J., Ge, X., Yu, J., Shan, S., Zhou, B., Song, S., et al. (2020). Human neutralizing antibodies elicited by SARS-CoV-2 infection. Nature *584*, 115–119.

Kreer, C., Zehner, M., Weber, T., Ercanoglu, M.S., Gieselmann, L., Rohde, C., Halwe, S., Korenkov, M., Schommers, P., Vanshylla, K., et al. (2020a). Longitudinal isolation of potent near-germline SARS-CoV-2-neutralizing antibodies from COVID-19 patients. Cell *182*, 843–854.e12.

Kreer, C., Gruell, H., Mora, T., Walczak, A.M., and Klein, F. (2020b). Exploiting B Cell receptor analyses to inform on HIV-1 vaccination strategies. Vaccines (Basel) *8*, 8.

Kreye, J., Reincke, S.M., Kornau, H.-C., Sánchez-Sendin, E., Max Corman, V., Liu, H., Yuan, M., Wu, N.C., Zhu, X., Lee, C.-C.D., et al. (2020). A SARS-CoV-2 neutralizing antibody protects from lung pathology in a COVID-19 hamster model. bioRxiv. https://doi.org/10.1101/2020.08.15.252320.

Lee, P.S., Ohshima, N., Stanfield, R.L., Yu, W., Iba, Y., Okuno, Y., Kurosawa, Y., and Wilson, I.A. (2014). Receptor mimicry by antibody F045-092 facilitates universal binding to the H3 subtype of influenza virus. Nat. Commun. *5*, 3614.

Lee, D.W., Khavrutskii, I.V., Wallqvist, A., Bavari, S., Cooper, C.L., and Chaudhury, S. (2017). BRILIA: Integrated Tool for High-Throughput Annotation and Lineage Tree Assembly of B-Cell Repertoires. Front. Immunol. *7*, 681.

Liu, H., Wu, N.C., Yuan, M., Bangaru, S., Torres, J.L., Caniels, T.G., van Schooten, J., Zhu, X., Lee, C.-C.D., Brouwer, P.J.M., et al. (2020a). Cross-neutralization of a SARS-CoV-2 antibody to a functionally conserved site is mediated by avidity. bioRxiv. https://doi.org/10.1101/2020.08.02.233536.

Liu, L., Wang, P., Nair, M.S., Yu, J., Rapp, M., Wang, Q., Luo, Y., Chan, J.F.-W., Sahi, V., Figueroa, A., et al. (2020b). Potent neutralizing antibodies against multiple epitopes on SARS-CoV-2 spike. Nature *584*, 450–456.

Lv, H., Wu, N.C., Tsang, O.T.-Y., Yuan, M., Perera, R.A.P.M., Leung, W.S., So, R.T.Y., Chan, J.M.C., Yip, G.K., Chik, T.S.H., et al. (2020). Cross-reactive Antibody Response between SARS-CoV-2 and SARS-CoV Infections. Cell Rep. *31*, 107725.

Marcou, Q., Mora, T., and Walczak, A.M. (2018). High-throughput immune repertoire analysis with IGoR. Nat. Commun. *9*, 561.

McKechnie, J.L., and Blish, C.A. (2020). The innate immune system: fighting on the front lines or fanning the flames of COVID-19? Cell Host Microbe *27*, 863–869.

Nielsen, S.C.A., and Boyd, S.D. (2018). Human adaptive immune receptor repertoire analysis-Past, present, and future. Immunol. Rev. *284*, 9–23.

Nielsen, S.C.A., Yang, F., Jackson, K.J.L., Hoh, R.A., Röltgen, K., Jean, G.H., Stevens, B.A., Lee, J.-Y., Rustagi, A., Rogers, A.J., et al. (2020). Human B cell clonal expansion and convergent antibody responses to SARS-CoV-2. Cell Host Microbe *28*, 516–525.e5.

Niu, X., Li, S., Li, P., Pan, W., Wang, Q., Feng, Y., Mo, X., Yan, Q., Ye, X., Luo, J., et al. (2020). Longitudinal Analysis of T and B Cell Receptor Repertoire Transcripts Reveal Dynamic Immune Response in COVID-19 Patients. Front. Immunol. *11*, 582010.

Nourmohammad, A., Otwinowski, J., Łuksza, M., Mora, T., and Walczak, A.M. (2019). Fierce selection and interference in B-Cell repertoire response to chronic HIV-1. Mol. Biol. Evol. *36*, 2184–2194.

Noy-Porat, T., Makdasi, E., Alcalay, R., Mechaly, A., Levy, Y., Bercovich-Kinori, A., Zauberman, A., Tamir, H., Yahalom-Ronen, Y., Israeli, M., et al. (2020). A panel of human neutralizing mAbs targeting SARS-CoV-2 spike at multiple epitopes. Nat. Commun. *11*, 4303.

Perera, R.A., Mok, C.K., Tsang, O.T., Lv, H., Ko, R.L., Wu, N.C., Yuan, M., Leung, W.S., Chan, J.M., Chik, T.S., et al. (2020). Serological assays for severe acute respiratory syndrome coronavirus 2 (SARS-CoV-2), March 2020. Euro Surveill. *25*, 2000421.

Pinto, D., Park, Y.-J., Beltramello, M., Walls, A.C., Tortorici, M.A., Bianchi, S., Jaconi, S., Culap, K., Zatta, F., De Marco, A., et al. (2020). Cross-neutralization of SARS-CoV-2 by a human monoclonal SARS-CoV antibody. Nature *583*, 290–295.

Pogorelyy, M.V., Minervina, A.A., Chudakov, D.M., Mamedov, I.Z., Lebedev, Y.B., Mora, T., and Walczak, A.M. (2018a). Method for identification of condition-associated public antigen receptor sequences. eLife *7*, e33050.

Pogorelyy, M.V., Minervina, A.A., Touzel, M.P., Sycheva, A.L., Komech, E.A., Kovalenko, E.I., Karganova, G.G., Egorov, E.S., Komkov, A.Y., Chudakov, D.M., et al. (2018b). Precise tracking of vaccine-responding T cell clones reveals convergent and personalized response in identical twins. Proc. Natl. Acad. Sci. USA *115*, 12704–12709.

Prabakaran, P., and Chowdhury, P.S. (2020). Landscape of non-canonical cysteines in human VH repertoire revealed by immunogenetic analysis. Cell Rep. *31*, 107831.

R Core Team (2020). R: A language and environment for statistical computing. R Foundation for Statistical Computing, Vienna, Austria. URL. https://www.R-project.org/.

Robbiani, D.F., Gaebler, C., Muecksch, F., Lorenzi, J.C.C., Wang, Z., Cho, A., Agudelo, M., Barnes, C.O., Gazumyan, A., Finkin, S., et al. (2020). Convergent antibody responses to SARS-CoV-2 in convalescent individuals. Nature *584*, 437–442.

Robins, H. (2013). Immunosequencing: applications of immune repertoire deep sequencing. Curr. Opin. Immunol. *25*, 646–652.

Rogers, T.F., Zhao, F., Huang, D., Beutler, N., Burns, A., He, W.-T., Limbo, O., Smith, C., Song, G., Woehl, J., et al. (2020). Isolation of potent SARS-CoV-2 neutralizing antibodies and protection from disease in a small animal model. Science *369*, 956–963.

Schultheiß, C., Paschold, L., Simnica, D., Mohme, M., Willscher, E., von Wenserski, L., Scholz, R., Wieters, I., Dahlke, C., Tolosa, E., et al. (2020). Next-generation sequencing of T and B cell receptor repertoires from COVID-19 patients showed signatures associated with severity of disease. Immunity *53*, 442–455.e4.

Sethna, Z., Isacchini, G., Dupic, T., Mora, T., Walczak, A.M., and Elhanati, Y. (2020). Population variability in the generation and selection of T-cell repertoires. PLoS Comput. Biol. *16*, e1008394.

Seydoux, E., Homad, L.J., MacCamy, A.J., Parks, K.R., Hurlburt, N.K., Jennewein, M.F., Akins, N.R., Stuart, A.B., Wan, Y.-H., Feng, J., et al. (2020a). Analysis of a SARS-CoV-2-infected individual reveals development of potent neutralizing antibodies with limited somatic mutation. Immunity *53*, 98–105.e5.

Seydoux, E., Homad, L.J., MacCamy, A.J., Parks, K.R., Hurlburt, N.K., Jennewein, M.F., Akins, N.R., Stuart, A.B., Wan, Y.-H., Feng, J., et al. (2020b). Characterization of neutralizing antibodies from a SARS-CoV-2 infected individual. bioRxiv. https://doi.org/10.1101/2020.05.12.091298.

Shi, R., Shan, C., Duan, X., Chen, Z., Liu, P., Song, J., Song, T., Bi, X., Han, C., Wu, L., et al. (2020). A human neutralizing antibody targets the receptor-binding site of SARS-CoV-2. Nature *584*, 120–124.

Storey, J.D. (2002). A direct approach to false discovery rates. J. R. Stat. Soc. Series B Stat. Methodol. *64*, 479–498.

Storey, J.D., and Tibshirani, R. (2003). Statistical significance for genomewide studies. Proc. Natl. Acad. Sci. USA *100*, 9440–9445.

Vabret, N., Britton, G.J., Gruber, C., Hegde, S., Kim, J., Kuksin, M., Levantovsky, R., Malle, L., Moreira, A., Park, M.D., et al.; Sinai Immunology Review Project (2020). Immunology of COVID-19: current state of the science. Immunity *52*, 910–941.

Vander Heiden, J.A., Yaari, G., Uduman, M., Stern, J.N.H., O'Connor, K.C., Hafler, D.A., Vigneault, F., and Kleinstein, S.H. (2014). pRESTO: a toolkit for processing high-throughput sequencing raw reads of lymphocyte receptor repertoires. Bioinformatics *30*, 1930–1932.

Wec, A.Z., Haslwanter, D., Abdiche, Y.N., Shehata, L., Pedreño-Lopez, N., Moyer, C.L., Bornholdt, Z.A., Lilov, A., Nett, J.H., Jangra, R.K., et al. (2020a). Longitudinal dynamics of the human B cell response to the yellow fever 17D vaccine. Proc. Natl. Acad. Sci. USA *117*, 6675–6685.

Wec, A.Z., Wrapp, D., Herbert, A.S., Maurer, D., Haslwanter, D., Sakharkar, M., Jangra, R.K., Dieterle, M.E., Lilov, A., Huang, D., et al. (2020b). Broad sarbecovirus neutralizing antibodies define a key site of vulnerability on the SARS-CoV-2 spike protein. bioRxiv. https://doi.org/10.1101/2020.05.15.096511.

World Health Organization (2021). Coronavirus disease (COVID-19) pandemic. https://www.who.int/emergencies/diseases/novel-coronavirus-2019.

Wrammert, J., Smith, K., Miller, J., Langley, W.A., Kokko, K., Larsen, C., Zheng, N.-Y., Mays, I., Garman, L., Helms, C., et al. (2008). Rapid cloning of high-affinity human monoclonal antibodies against influenza virus. Nature *453*, 667–671.

Wu, Y.-C., Kipling, D., and Dunn-Walters, D. (2015). Assessment of B cell repertoire in humans. Methods Mol. Biol. *1343*, 199–218.

Wu, J.T., Leung, K., Bushman, M., Kishore, N., Niehus, R., de Salazar, P.M., Cowling, B.J., Lipsitch, M., and Leung, G.M. (2020a). Estimating clinical severity of COVID-19 from the transmission dynamics in Wuhan, China. Nat. Med. *26*, 506–510.

Wu, Y., Wang, F., Shen, C., Peng, W., Li, D., Zhao, C., Li, Z., Li, S., Bi, Y., Yang, Y., et al. (2020b). A noncompeting pair of human neutralizing antibodies block COVID-19 virus binding to its receptor ACE2. Science *368*, 1274–1278.

Yuan, M., Wu, N.C., Zhu, X., Lee, C.D., So, R.T.Y., Lv, H., Mok, C.K.P., and Wilson, I.A. (2020). A highly conserved cryptic epitope in the receptor binding domains of SARS-CoV-2 and SARS-CoV. Science *368*, 630–633.

Zhou, D., Duyvesteyn, H.M.E., Chen, C.-P., Huang, C.-G., Chen, T.-H., Shih, S.-R., Lin, Y.-C., Cheng, C.-Y., Cheng, S.-H., Huang, Y.-C., et al. (2020). Structural basis for the neutralization of SARS-CoV-2 by an antibody from a convalescent patient. Nat. Struct. Mol. Biol. *27*, 950–958.

Zost, S.J., Gilchuk, P., Chen, R.E., Case, J.B., Reidy, J.X., Trivette, A., Nargi, R.S., Sutton, R.E., Suryadevara, N., Chen, E.C., et al. (2020). Rapid isolation and profiling of a diverse panel of human monoclonal antibodies targeting the SARS-CoV-2 spike protein. Nat. Med. *26*, 1422–1427.

STAR★METHODS

KEY RESOURCES TABLE

REAGENT or RESOURCE	SOURCE	IDENTIFIER
**Antibodies**		
Goat anti-Human IgG (H+L) Secondary Antibody, HRP	Thermo Fisher Scientific	RRID:AB_2535582
**Biological samples**		
Plasma from SARS-CoV-2 patients	Hospital Authority of Hong Kong	N/A
Plasma from healthy donors	Hong Kong Red Cross	N/A
**Chemicals, peptides, and recombinant proteins**		
PBS	Thermo Fisher	10010023
Trypsin	Thermo Fisher	15050065
Tween 20	Thermo Fisher	BP337-500
Ficoll-Paque Plus medium	GE Healthcare	17144002
RPMI 1640 Medium	Thermo Fisher	11879020
Fetal Bovine Serum	Thermo Fisher	10099141
Dimethyl Sulfoxide (DMSO)	Sigma	472301
Chonblock blocking/sample dilution ELISA buffer	Chondrex Inc	9068
HRP substrate	Ncm TMB One	M30100
**Critical commercial assays**		
RNeasy Mini isolation kit	QIAGEN	74106
Proto- Script® II Reverse Transcriptase kit	New England Biolabs	M0368S
Phusion® High-Fidelity DNA Polymerase	New England Biolabs	M0530S
QIAquick Gel Extraction Kit	QIAGEN	28704
Bac-to-Bac® Baculovirus Expression System	Thermo Fisher	10359-016
Plasma Cell Isolation Kit II, human	Miltenyi Biotec	130-093-628
B Cell Isolation Kit II, human	Miltenyi Biotec	130-091-151
**Deposited data**		
SARS-CoV-2 spike protein sequence	NCBI Reference Sequence	YP_009724390.1
SARS-CoV spike protein sequence	GenBank	ABF65836.1
High-throughput B cell receptor sequencing data (bulk)	NCBI BioProject	PRJNA645245
High-throughput B cell receptor sequencing data (plasma)	NCBI BioProject	PRJNA679920
Raw and annotated sorted single cell sequencing data	GitHub	https://github.com/StatPhysBio/covid-bcr/tree/master/singlecell_data
**Cell lines**		
Sf9 cells	ATCC	CRL-1711
High Five cells	Thermo Fisher Scientific	B85502
**Oligonucleotides**		
Primers for PCR	Integrated DNA Technologies	N/A
**Recombinant DNA**		
pFastBac-SARS-CoV-2 spike ectodomain	Lv et al., 2020	N/A
pFastBac-SARS-CoV spike ectodomain	Lv et al., 2020	N/A
pFastBac-SARS-CoV-2 RBD	Lv et al., 2020	N/A
pFastBac-SARS-CoV RBD	Lv et al., 2020	N/A

(*Continued on next page*)

Continued		
REAGENT or RESOURCE	SOURCE	IDENTIFIER
Software and algorithms		
R, 4.0.4	R Core Team, 2020	https://www.r-project.org
pRESTO, 0.5.13	Vander Heiden et al., 2014	https://presto.readthedocs.io/en/stable/overview.html
abstar, 0.3.5	Briney and Burton, 2018	https://github.com/briney/abstar
IGoR, 1.4	Marcou et al., 2018	https://github.com/qmarcou/IGoR
SONIA, 0.45	Sethna et al., 2020	https://github.com/statbiophys/SONIA
covid-bcr		https://github.com/StatPhysBio/covid-bcr
Other		
HyClone insect cell culture medium	GE Healthcare	SH30280.03
DMEM	Thermo Fisher Scientific	11965-092
Nunc MaxiSorp ELISA plate	Thermo Fisher Scientific	44-2404-21
Ni-NTA Superflow	QIAGEN	30450
FuGENE HD	Promega	E2311
DH10Bac competent cells	Thermo Fisher Scientific	10361012
Trypan Blue Solution, 0.4%	Thermo Fisher	15250061

RESOURCE AVAILABILITY

Lead contact

Further information and requests for resources, reagents, code and the data should be directed to and will be fulfilled by the Lead Contact, Armita Nourmohammad (armita@uw.edu).

Materials availability

All reagents generated in this study are available from the Lead Contact with a completed Materials Transfer Agreement.

Data and code availability

The accession numbers for the BCR repertoire raw fastq data and single-cell data reported in this paper are: BioProject: PRJNA645245, PRJNA679920.

https://www.ncbi.nlm.nih.gov/bioproject/PRJNA645245

All code for data processing and statistical analysis can be found at: https://github.com/StatPhysBio/covid-BCR

EXPERIMENTAL MODEL AND SUBJECT DETAILS

Cell lines

Sf9 cells (*Spodoptera frugiperda* ovarian cells, female, ATCC catalog no. CRL-1711) and High Five cells (*Trichoplusia ni* ovarian cells, female; Thermo Fisher Scientific, Waltham, United States (US), catalog number: B85502) were maintained in HyClone (GE Health Care, Chicago, US) insect cell culture medium.

Patients and samples

Specimens of heparinized blood were collected from the RT-PCR-confirmed patients with COVID-19 at the Infectious Disease Centre of the Princess Margaret Hospital, Hong Kong. The study was approved by the institutional review board of the Hong Kong West Cluster of the Hospital Authority of Hong Kong (approval number: UW20-169). All study procedures were performed after informed consent was obtained. Day 1 of clinical onset was defined as the first day of the appearance of clinical symptoms. The severity of the COVID-19 cases was classified based on the adaptation of the Sixth Revised Trial Version of the Novel Coronavirus Pneumonia Diagnosis and Treatment Guidance. The severity of the patients was categorized as follows: Mild - no sign of pneumonia on imaging, mild clinical symptoms; Moderate - fever, respiratory symptoms and radiological evidence of pneumonia; Severe - dyspnea, respiratory frequency > 30/min, blood oxygen saturation 93%, partial pressure of arterial oxygen to fraction of inspired oxygen ratio < 300, and/or lung infiltrates > 50% within 24 to 48 hours; Critical - respiratory failure, septic shock, and/or multiple organ dysfunction or failure or death. For details on age, sex, and severity of each individual, see Data S1.

METHOD DETAILS

PBMC isolation

The blood samples were first centrifuged at 3000 xg for 10 minutes at room temperature for plasma collection. The remaining blood was diluted with equal volume of PBS buffer, transferred onto the Ficoll-Paque Plus medium (GE Healthcare), and centrifuged at 400 xg for 20 minutes. Peripheral Blood Mononuclear Cells (PBMC) samples were then collected and washed with cold RPMI-1640 medium for three times. The isolated PBMC samples were finally stored at cell freezing solution (10% DMSO + 90% FBS) and kept in −80°C until used.

RNA extraction and reverse transcription

Total RNA was extracted from 5×10^5 PBMC using the RNeasy Mini isolation kit (QIAGEN) according to the manufacturer's protocol. Reverse transcription of the RNA samples was performed using the Proto- Script® II Reverse Transcriptase kit (New England Biolabs, NEB) with random hexamer primers according to the manufacturer's protocol. The thermal cycling conditions were designed as follows: 25°C for 5 minutes, 42°C for 60 minutes, and 80°C for 5 minutes. The resulting cDNA samples were stored in 80°C freezer before PCR was performed.

Amplification of B cell repertoire from the samples by PCR

The cDNA samples were used as a template to amplify the antibody IgG heavy chain gene with six FR1-specific forward primers and one constant region-specific reversed primer using the Phusion® High-Fidelity DNA Polymerase. The primer sequences were the same as previously described (Wu et al., 2015); primer sequences are listed in Table S1. The thermal cycling conditions were set as follows: 98°C for 30 s; 30 cycles of 98°C for 10 s, 58°C for 15 s, and 72°C for 30 s; and 72°C for 10 minutes. Then 10 ng of the PCR product was used as a template for the next round of gene amplification with sample-specific barcode primers. The thermal cycling conditions were set as follow: 98°C for 3 min; 30 cycles of 98°C for 10 s, 58°C for 15 s, and 72°C for 15 s; and a final extension at 72°C for 10 min using Phusion® High-Fidelity DNA Polymerase. The PCR product was purified by QIAquick Gel Extraction Kit (QIAGEN), and quantified by NanoDrop Spectrophotometers (Thermofisher).

Protein expression and purification

The receptor-binding domain (RBD, residues 319–541) and N-terminal domain (NTD, residues 14 to 305) of the SARS-CoV-2 spike protein (GenBank: QHD43416.1) as well as the RBD (residues 306-527) and NTD (residues 14-292) of SARS-CoV-1 spike protein (GenBank: ABF65836.1) were cloned into a customized pFastBac vector (Lv et al., 2020; Wec et al., 2020b). The RBD and NTD constructs were fused with an N-terminal gp67 signal peptide and a C-terminal His_6 tag. Recombinant bacmid DNA was generated using the Bac-to-Bac system (Life Technologies, Thermo Fisher Scientific). Baculovirus was generated by transfecting purified bacmid DNA into Sf9 cells using FuGENE HD (Promega, Madison, US) and subsequently used to infect suspension cultures of High Five cells (Life Technologies) at a multiplicity of infection (moi) of 5 to 10. Infected High Five cells were incubated at 28 °C with shaking at 110 rpm for 72 h for protein expression. The supernatant was then concentrated using a Centramate cassette (10 kDa molecular weight cutoff for RBD, Pall Corporation, New York, USA). RBD and NTD proteins were purified by Ni-NTA Superflow (QIAGEN, Hilden, Germany), followed by size exclusion chromatography and buffer exchange to phosphate-buffered saline (PBS).

$CD38^+$ plasma B cell enrichment

$CD38^+$ plasma B cells were isolated from the PBMC samples by performing two subsequent magnetic separation steps according to the manufacturer's protocol (Plasma Cell Isolation Kit II, human, Miltenyi Biotec). Briefly, non-plasma B cells are labeled with magnetic beads combined with cocktail antibodies and separated using the MACS column. Then, $CD38^+$ plasma B cells are directly labeled with CD38 MicroBeads and isolated from the pre-enriched B cell pool. Purified $CD38^+$ plasma B cells were eluted and washed in PBS containing 2% (v/v) fetal bovine serum (FBS) and kept for the following RNA isolation step. In order to test the purity of the $CD38^+$ plasma B cells, we also added staining antibodies and 10 μL of Anti-human CD19-BV510 (BioLegend) and CD38-PE-Cy7 (BioLegend) and incubated them for 15 minutes in the dark in the refrigerator (2-8°C). Cells were finally fixed with 4% PFA for 20 minutes on ice. The stained samples were acquired by flow cytometry on a FACS Attune (Invitrogen) and analyzed with FlowJo software (Figure S1).

RBD and NTD protein specific binding B cell enrichment

B cells were enriched from the PBMC samples according to the manufacture's protocol (B Cell Isolation Kit II, human, Miltenyi Biotec). Briefly, non-B cells are labeled with a cocktail of biotin-conjugated antibodies and separated by the MACS column. Purified B cells were eluted and kept in the PBS buffer with 2% (v/v) FBS. The enriched B cells were then incubated with 2 μg Biotin-RBD or NTD protein for 30 min at 4°C. After incubation, Anti-Biotin MicroBeads were added and incubated for 30 min. RBD and NTD specific bead binding B cells were washed and eluted in PBS and stored on ice until use. In order to test the purity of the RBD- or NTD-specific B cells, we also added staining antibodies, 10 μL of Anti-human CD19-BV510 (BioLegend), and 2 μg of SARS-CoV-2 RBD-PE or NTD-PE and incubated them for one hour in the dark in the refrigerator (2-8°C). Cells were finally fixed with 4% PFA for 20 minutes on ice. The stained samples were acquired by flow cytometry on a FACS Attune (Invitrogen) and analyzed with FlowJo software (Figure S1).

Single B cell 5′ mRNA and VDJ sequencing

After RBD or NTD specific B cells enrichment, cells were counted by using 0.4% (w/v) trypan blue stain solution in the microscope and directly loaded on the 10X Chromium™ Single Cell A Chip. Then single B cell lysis and RNA first-strand synthesis were carried out following the 10X Chromium™ Single Cell 5′ Library & Gel Bead Kit protocol. The RNA sample were used for the next step B cell VDJ library construction following the Chromium™ Single Cell V(D)J Enrichment Kits protocol. VDJ library sequencing was performed on a NovaSeq PE150 and the sequencing data were processed by Cell Ranger.

ELISA

A 96-well enzyme-linked immunosorbent assay (ELISA) plate (Nunc MaxiSorp, Thermo Fisher Scientific) was first coated overnight with 100 ng per well of purified recombinant protein in PBS buffer. The plates were then blocked with 100 μL of Chonblock blocking/sample dilution ELISA buffer (Chondrex Inc, Redmon, US) and incubated at room temperature for 1 h. Each human plasma sample was diluted to 1:100 in Chonblock blocking/sample dilution ELISA buffer. Each sample was then added into the ELISA plates for a two-hour incubation at 37°C. After extensive washing with PBS containing 0.1% Tween 20, each well in the plate was further incubated with the anti-human IgG secondary antibody (1:5000, Thermo Fisher Scientific) for 1 hour at 37°C. The ELISA plates were then washed five times with PBS containing 0.1% Tween 20. Subsequently, 100 μL of HRP substrate (Ncm TMB One; New Cell and Molecular Biotech Co. Ltd, Suzhou, China) was added into each well. After 15 min of incubation, the reaction was stopped by adding 50 μL of 2 M H_2SO_4 solution and analyzed on a Sunrise (Tecan, Männedorf, Switzerland) absorbance microplate reader at 450 nm wavelength.

BCR preprocessing

We used a similar procedure for processing of the bulk and the plasma B cell receptor repertoires. For initial processing of the raw reads, we used pRESTO (version 0.5.13) (Vander Heiden et al., 2014) to assemble paired-end reads, remove sequences with a mean quality score less than 30, mask primer subsequences, and collapse duplicate sequences into unique sequences. The small fraction of paired-end reads that overlapped were assumed to be anomalous and were discarded from the analysis. Additionally, after preprocessing with pRESTO, we discarded unique reads that contained ambiguous calls (N's) in their receptor sequence.

BCR error correction

We performed two rounds of error correction on sequences that passed the quality control check. In the first round, we clustered singletons and other low-frequency sequences into larger sequences if they were similar in sequence. The intent of this round was to correct for sequencing errors (e.g., from reverse transcription of mRNA to cDNA) that caused large abundance clones to be split into many similar sequences. We used two parameters: $\Delta_r = 1.0$, the marginal Hamming distance tolerance per decade in log-ratio abundance (each $\log_{10}$ unit allowing Δ_r additional sequence differences), and $\Delta_a = 1.0$, the marginal abundance tolerance of clusterable sequences per decade in log-ratio abundance (each $\log_{10}$ unit allowing abundance Δ_a higher as clusterable). For example, a sequence with abundance a_1 and a Hamming distance d away from a higher abundance sequence with abundance a_2 was absorbed into the latter only if $d \le \Delta_r \log_{10} \frac{a_2}{a_1}$ and $a_1 \le \Delta_a \log_{10} \frac{a_2}{a_1}$. We used the output of this first round as input for the second round of error correction, in which we more aggressively target correction of reverse transcriptase errors. In the second round, we used two different parameters to assess sequence similarity: $d_{\text{thresh}} = 2.0$, the Hamming distance between sequences, and $a_{\text{thresh}} = 1.0$, the ratio of sequence abundances. A sequence with abundance a_1 and a Hamming distance d away from a sequence of larger abundance a_2 was absorbed into the latter only if $d \le d_{\text{thresh}}$ and the ratio of the sequence abundances was greater than a_{thresh}, i.e., $\frac{a_2}{a_1} \ge a_{\text{thresh}}$. This round of error correction allows much larger abundance sequences to potentially be clustered than is possible in the first round. For both of the above steps, we performed clustering greedily and approximately by operating on sequences sorted by descending abundance, assigning the counts of the lower abundance sequence to the higher abundance one iteratively.

After error correction, the sequences still contained a large number of singletons, i.e., sequences with no duplicates (Data S1). We discarded these singletons from all analyses that relied on statistics of unique sequences (i.e., the results presented in Figures S2A–S2C and S3E–S3G).

BCR annotation

For each individual, error-corrected sequences from all time points and technical replicates were pooled and annotated by abstar (version 0.3.5) (Briney and Burton, 2018). We processed the output of abstar, which included the estimated IGHV gene/allele, IGHJ gene/allele, location of the HCDR3 region, and an inferred naive sequence (germline before hypermutation). Sequences which had indels outside of the HCDR3 were discarded. We partitioned the sequences into two sets: productive BCRs, which were in-frame and had no stop codons, and unproductive BCRs, which were out-of-frame.

Unproductive BCRs

Due to a larger sequencing depth in healthy individuals, we were able to reconstruct relatively large unproductive BCR lineages. Unproductive sequences are BCRs that are generated but, due to a frameshift or insertion of stop codons, are never expressed. These BCRs reside with productive (functional) BCRs in a nucleus and undergo hypermutation during B cell replication and, therefore, provide a suitable null expectation for generation of BCRs in immune repertoires.

Clonal lineage reconstruction

To identify BCR clonal lineages, we first grouped sequences by their assigned IGHV gene, IGHJ gene, and HCDR3 length and then used single-linkage clustering with a threshold of 85% Hamming distance. A similar threshold has been suggested previously by Gupta et al. (2017) to identify BCR lineages. Defining size as the sum of the number of unique sequences per time point within a lineage, clusters of size smaller than three were discarded from most analyses. They were retained only for training receptor generation and selection models and were not discarded in the sharing analysis only if the progenitor of that small cluster was also a progenitor of a cluster of size at least three in another patient. For each cluster, there may have been multiple inferred naive sequences, as this was an uncertain estimate. Therefore, the most common naive sequence was chosen to be the naive progenitor of the lineage. When the most common naive sequence of a productive lineage contained a stop codon, the progenitor of the lineage was chosen iteratively by examining the next most common naive sequence until it did not contain any stop codons. If all inferred naive sequences in a productive lineage had a stop codon, that lineage was discarded from the analysis. Data S1 shows the statistics of constructed clonal lineages in each individual for the bulk repertoire and combined bulk+plasma B cell repertoire, respectively.

Mapping of single-cell data onto reconstructed clonal lineages

Like the repertoire datasets, the single-cell sequences were annotated by abstar (Briney and Burton, 2018). For each receptor acquired by single-cell sequencing, we identified a subset of reconstructed clonal lineages from the bulk repertoire which had identical HCDR3 length as the sequence and which also had an IGHV gene which was 90% similar to that of the single-cell receptor. This flexibility in V-gene choice would identify functionally homologous receptors and associate a receptor to a lineage with a sequence divergence in the V-segment, compatible with the expectation under somatic hypermutations (Lee et al., 2017). A single-cell sequence was matched to a reconstructed clonal lineage from this subset if its HCDR3 could be clustered with other members of the lineages, using single-linkage clustering with a similarity threshold of 85% Hamming distance (similar to the criteria for lineage reconstruction for bulk repertoires).

Inference of generation probability and selection for BCRs

We used IGoR (version 1.4) (Marcou et al., 2018) to obtain a model of receptor generation. This model characterized the probability of generation $P_{\text{gen}}(\sigma)$ of a receptor dependent on the features of the receptor, including the IGHV, IGHD, and IGHJ genes and the deletion and insertion profiles at the VD and DJ junctions. To characterize the parameters of this model, we trained IGoR on the progenitors of unproductive lineages, regardless of size, pooled from the bulk repertoire of all individuals, restricted to progenitors whose HCDR3 began with a cysteine and ended with a tryptophan. For consistency with our receptor annotations based on abstar, we used abstar's genomic templates and the HCDR3 anchors of abstar's reference genome as inputs for IGoR's genomic templates and HCDR3 anchors. $P_{\text{gen}}(\sigma)$ distributions of the healthy and COVID-19 cohorts in this study are shown in Figure S4A.

We used SONIA (version 0.45) (Sethna et al., 2020) to infer a selection model for progenitors of productive clonal lineages. The SONIA model evaluated selection factors q to characterize the deviation in the probability $P_{\text{post}}(\sigma)$ to observe a functional sequence in the periphery from the null expectation based on the generation probability $P_{\text{gen}}(\sigma) : P_{\text{post}}(\sigma) = \frac{1}{Z} P_{\text{gen}}(\sigma) e^{\Sigma_{f:\text{features}} q_f(\sigma)}$, where Z is the normalization factor and $q_f(\sigma)$ are selection factors dependent on the sequence features f. These sequence features include IGHV-gene and IGHJ-gene usages and HCDR3 length and amino acid composition (Sethna et al., 2020).

In our analysis, we used the SONIA left-right model with independent IGHV- and IGHJ-gene usages (Sethna et al., 2020). We used the output from IGoR (Marcou et al., 2018) as the receptor generation model for SONIA. We trained four cohort-specific selection models on progenitors of productive lineages, regardless of size, pooled from the bulk repertoire of all individuals within a cohort, restricted to progenitors whose HCDR3 began with a cysteine and ended with a tryptophan. 150 epochs, L_2 regularization with strength 0.001, and 500,000 generated sequences were used to train each SONIA model. Figure 3 shows the distributions for the probabilities of observing productive receptors sampled from each cohort $P_{\text{post}}(\sigma)$. A selection model was also trained on all the productive lineage progenitors in the GRP dataset (Briney et al., 2019) and used 5,000,000 generated sequences, keeping the other parameters unchanged. We refrain from comparing directly $P_{\text{post}}(\sigma)$ associated with GRP BCRs to BCRs in this study due to experimental differences.

It should be noted that the (pre-selection) generation model $P_{\text{gen}}(\sigma)$ inferred by IGoR (Marcou et al., 2018) is robust to sequence errors due to experimental errors or hypermutations in the IgG repertoires. However, hypermutations in BCRs could introduce errors in inference of selection models and estimation of receptor probabilities by SONIA (Sethna et al., 2020). Therefore, we have restricted our selection analyses to only the inferred progenitors of clonal lineages. Although the inferred progenitors of lineages can still deviate from the *true* (likely IgM naive) progenitors, the selection models inferred from *ensembles* of inferred progenitors in IgG repertoires seem to be comparable to the models inferred from the IgM repertoires (M. Ruiz Ortega, personal communication). The resulting selection models, trained on either true or inferred progenitors, reflect preferences for sequence features of unmutated receptors, including IGHV- and IGHJ- genes and HCDR3 length and composition, but they do not account for the hypermutation preferences that may distinguish one cohort from another.

Characterizing the robustness of selection inference

To test the sensitivity of the inferred selection models on the size of the training sets, we down-sampled the receptor data of each COVID-19 cohort to a size comparable to the smallest cohort, i.e., the healthy repertoire sequenced in this study. This down-sampling

resulted in two independent training datasets for the mild COVID-19 cohort, 13 independent training datasets for the moderate COVID-19 cohort, and three independent training datasets for the severe COVID-19 cohort. Though this down-sampling resulted in over 400 independent training datasets for the GRP, we elected to use only 15. We then inferred a separate selection model with SONIA for each of these training datasets and used each model to evaluate the receptor log-probabilities $\log_{10} P_{\text{post}}(\sigma)$ for a set of 500,000 generated receptors. The evaluated probabilities are strongly correlated between models inferred from the down-sampled data in each cohort, with a Pearson correlation of $r > 0.99$ and p value = 0 (p value is smaller than machine precision); see Figures S4C–S4F.

We used a similar approach to compare the selection model inferred from the healthy repertoires sequenced in this study and the GRP study (Briney et al., 2019). Figure S4B shows that, using the model inferred with our healthy repertoire and 30 down-sampled independently inferred selection models using the GRP dataset, the evaluated log-probabilities $\log_{10} P_{\text{post}}(\sigma)$ based on these two datasets are strongly correlated, with a Pearson correlation of $r > 0.99$ and p value = 0 (p value is smaller than machine precision); see Figure S4B.

Characterizing repertoire diversity

We quantified the diversity of each cohort by evaluating the entropy of receptor sequences in each cohort. Entropy can be influenced by the size of the training dataset for the selection models. To produce reliable estimates of repertoires' diversities (and entropies), we used the procedure described above to learn independent selection models for subsampled repertoires in each cohort. We then used the inferred IGoR and SONIA models to generate 500,000 synthetic receptors based on each of the subsampled, cohort-specific models. We evaluated cohort entropies H as the expected log-probabilities to observe a functional sequence in the respective cohort: $H = -\sum_{\sigma} P_{\text{post}}(\sigma) \log P_{\text{post}}(\sigma)$; the estimates based on the generated receptors are reported in the main text. The error bars reported for these entropy estimates are due to variations across the inferred models in each cohort.

For comparison, we also evaluated the entropy estimated on the repertoire data in each cohort, which showed a similar pattern to the estimates from the generated cohorts (in the main text). Specifically, the entropy of BCR repertoires estimated from the data follows: 39.8 ± 0.3 bits in healthy individuals, 41.9 ± 0.7 bits for patients in the mild cohort, 42.7 ± 0.3 bits for patients in the moderate cohort, and 42.9 ± 0.5 for patients in the severe cohort. The error bars indicate the standard error due to differences among individuals within a cohort.

Comparing selection between repertoires of cohorts

Selection models enable us to characterize the sequence features of immune repertoires that differ between cohorts. We evaluated the Jensen-Shannon divergence $D_{\text{JS}}(r, r')$ between the distribution of repertoires r and r', P^{r}_{post} and $P^{r'}_{\text{post}}$, defined as

$$D_{\text{JS}}(r, r') = \frac{1}{2} \sum_{\sigma:\ \text{sequences}} P^{r}_{\text{post}}(\sigma) \log \frac{P^{r}_{\text{post}}(\sigma)}{\left(P^{r}_{\text{post}}(\sigma) + P^{r'}_{\text{post}}(\sigma)\right)/2} + \frac{1}{2} \sum_{\sigma:\ \text{sequences}} P^{r'}_{\text{post}}(\sigma) \log \frac{P^{r'}_{\text{post}}(\sigma)}{\left(P^{r}_{\text{post}}(\sigma) + P^{r'}_{\text{post}}(\sigma)\right)/2}$$

$$= \frac{1}{2} \sum_{\sigma:\ \text{sequences}} P^{r}_{\text{post}}(\sigma) \log \frac{2\,Q^{r}(\sigma)}{Q^{r}(\sigma) + Q^{r'}(\sigma)} + \frac{1}{2} \sum_{\sigma:\ \text{sequences}} P^{r'}_{\text{post}}(\sigma) \log \frac{2\,Q^{r'}(\sigma)}{Q^{r}(\sigma) + Q^{r'}(\sigma)}$$

where we used the relationship between a receptor's generation probability $P_{\text{gen}}(\sigma)$ and its probability after selection $P^{r}_{\text{post}}(\sigma)$, using the inferred selection factor $Q^{r}(\sigma) = \frac{1}{Z} e^{\sum_{f:\text{features}} q^{r}_{f}(\sigma)}$ in repertoire r: $P^{r}_{\text{post}}(\sigma) = P_{\text{gen}}(\sigma)\, Q^{r}(\sigma)$. The Jensen-Shannon divergence $D_{\text{JS}}(r, r')$ is a symmetric measure of distance between two repertoires, which we can calculate using their relative selection factors (Isacchini et al., 2021). Figure 3 shows the expected partial Jensen-Shannon divergences evaluated over five independent realizations of 100,000 generated sequences for each partial selection model. The error bars show the variations of these estimates (i.e., standard deviation) over the five independent realizations in this procedure.

Clonal lineage expansion

We studied clonal lineage expansion of BCR repertoires in individuals that showed an increase in the binding level (OD_{450}) of their plasma to SARS-CoV-2 (RBD) during infection (Figures 4A and S5): patients 2, 3, 4, 5, 6, 7, 9, 10, 11, 13, 14. Other individuals showed no increase in IgG binding to SARS-CoV-2 (RBD), either due to already high levels of binding at early time points or to natural variation and noise (Figure S5). Our expansion test compared two time points. Therefore, for individuals with three time points, we combined data from different time points such that the separated times coincided with larger changes in binding levels (OD_{450}). Specifically, we combined the last two time points for patients 2 and 7 and the first two time points for patient 9. In addition, we combined the technical replicates at the same time point and filtered out small lineages with size less than three, where size was defined as the sum of the amount of unique sequences per time-point within a lineage.

To test for expansion, we compared lineage abundances (i.e., total number of reads in a lineage) between early and late time points. Many lineages appeared only in one time point due to the sparse sampling of clonal lineages and the cells that generate them (Figure S6). Therefore, we tested for expansion only for lineages that had nonzero abundances at both time points.

Our expansion test relied on comparing the relative abundance of a given lineage with other lineages. However, due to primer-specific amplification biases, abundances were not comparable between reads amplified with different primers. Therefore, in our analysis we only compare a lineage with all other lineages that were amplified with the same primer.

We applied a hypergeometric test (Fisher's exact test) to characterize significance of abundance fold change for a focal lineage. A similar method was used to study clonal expansion in TCRs (DeWitt et al., 2015). For each focal clonal lineage i (in a given individual), we defined a 2 ×2 contingency matrix C,

$$C = \begin{pmatrix} n_i^{\text{early}} & N_{/i}^{\text{early}} \\ n_i^{\text{late}} & N_{/i}^{\text{late}} \end{pmatrix}$$

where n_i^{early} and n_i^{late} are the abundances of the focal lineage at the early and late time, and $N_{/i}^{\text{early}}$ and $N_{/i}^{\text{late}}$ are the total abundances of all reads (with the same primer) minus those from lineage i at the early and late times. The ratio $\frac{n_i^{late}}{n_i^{early}} \Big/ \frac{N_{/i}^{late}}{N_{/i}^{early}}$ describes the fold change, or odds ratio, of lineage i relative to the rest of the reads in the same primer group. Based on the contingency matrix C, one-sided p values for Fisher's exact test were calculated using the "fisher.test" function in R version 4.0. Fold change and p values are shown in Figure S6G.

To determine a significance threshold for the Fisher's exact test, we examined the technical replicate data from samples collected from the same time point in each individual because we did not expect any significant expansion among technical replicates. We performed the expansion test on pairs of technical replicates (Figure S6C) and compared the empirical cumulative distributions of the time point and replicate expansion data (Figures S6E and S6F) (Storey, 2002; Storey and Tibshirani, 2003). We chose a p value threshold of 10^{-300}, where there were 12.3 as many significant expansions as in the replicate data, and therefore the false discovery rate was approximately $1/(1 + 12.3) = 0.075$.

Significance of BCR sharing among individuals

The probability that receptor σ is shared among a given number of individuals due to convergent recombination can be evaluated based on the probability to observe a receptor in the periphery $P_{\text{post}}(\sigma)$, the size of the cohort M, and the size of the repertoire (sequence sample size) N. First, we evaluated the probability $\rho(\sigma; N)$ that receptor σ with probability $P_{\text{post}}(\sigma)$ appears at least once in a sample of size N,

$$\rho(\sigma; N) = 1 - (1 - P_{\text{post}}(\sigma))^N \simeq 1 - e^{-NP_{\text{post}}}$$

The probability that receptor σ is shared among m individuals out of a cohort of M individuals, each with a (comparable) sample size N, follows the binomial distribution,

$$P_{\text{share}}(\sigma; m, M, N) = \binom{M}{m} [\rho(\sigma; N)]^m [1 - \rho(\sigma; N)]^{M-m}$$

We aimed to identify shared receptors that were outliers such that their probability of sharing is too small to be explained by convergent recombination or other biases in the data. To do so, we identified the receptors with the smallest sharing probabilities P_{share} and found a threshold of P_{post} (dashed lines in Figures 5 and S7) at the 2% quantile of P_{share} in the data. Specifically, since P_{share} is a function of P_{post} and m (number of individuals sharing), for each m we solved for P_{post} such that $P_{\text{share}} = c$, and tuned the constant c such that only 2% of the data lay below P_{share}. This was a conservative choice to identify the rare shared outliers in the data.

QUANTIFICATION AND STATISTICAL ANALYSIS

Differences in the mean HCDR3 lengths and $\log_{10}$ relative read abundance in the plasma B cell repertoire for expanded and non-expanded lineages were studied by ordinary one-way ANOVA tests using SciPy 1.5 and python 3.8.5. Results can be found in the captions of Figures 2 and S2 and in the caption of Figure 4, respectively. The Pearson correlation coefficients and p values associated with testing for non-correlation for the correspondences between the bulk and plasma repertoires of patients, results shown in the legend of Figure S3A, and between the $\log_{10}P_{\text{post}}$ of independently trained SONIA models, results shown in plots and the caption of Figure S4, were found using SciPy 1.5. Fisher exact tests for the expansion analyses were calculated using the "fisher.test" function in R version 4.0 and in Python using the fisher module found here https://github.com/brentp/fishers_exact_test. The details of how the Fisher exact tests were constructed can be found in Methods, and the results are shown in Figures 4, 5, and S6. IGoR 1.4 was used to infer a baseline generation model, and SONIA 0.45 was used to infer a selection model. Details on how both were used can be found in Methods. Binomial sampling p values were obtained using SciPy 1.5. Jensen-Shannon divergences, entropy estimates, and the statistical analysis for identifying rare receptors were detailed in Methods and were developed in-house and can be found in the GitHub repository for this paper at https://github.com/StatPhysBio/covid-BCR. Where appropriate, it is indicated in both the main text and figure captions when standard deviation or standard error of the mean are used.

Cell Reports, Volume *35*

Supplemental information

Dynamics of B cell repertoires and emergence of cross-reactive responses in patients with different severities of COVID-19

Zachary Montague, Huibin Lv, Jakub Otwinowski, William S. DeWitt, Giulio Isacchini, Garrick K. Yip, Wilson W. Ng, Owen Tak-Yin Tsang, Meng Yuan, Hejun Liu, Ian A. Wilson, J.S. Malik Peiris, Nicholas C. Wu, Armita Nourmohammad, and Chris Ka Pun Mok

Supplementary Information

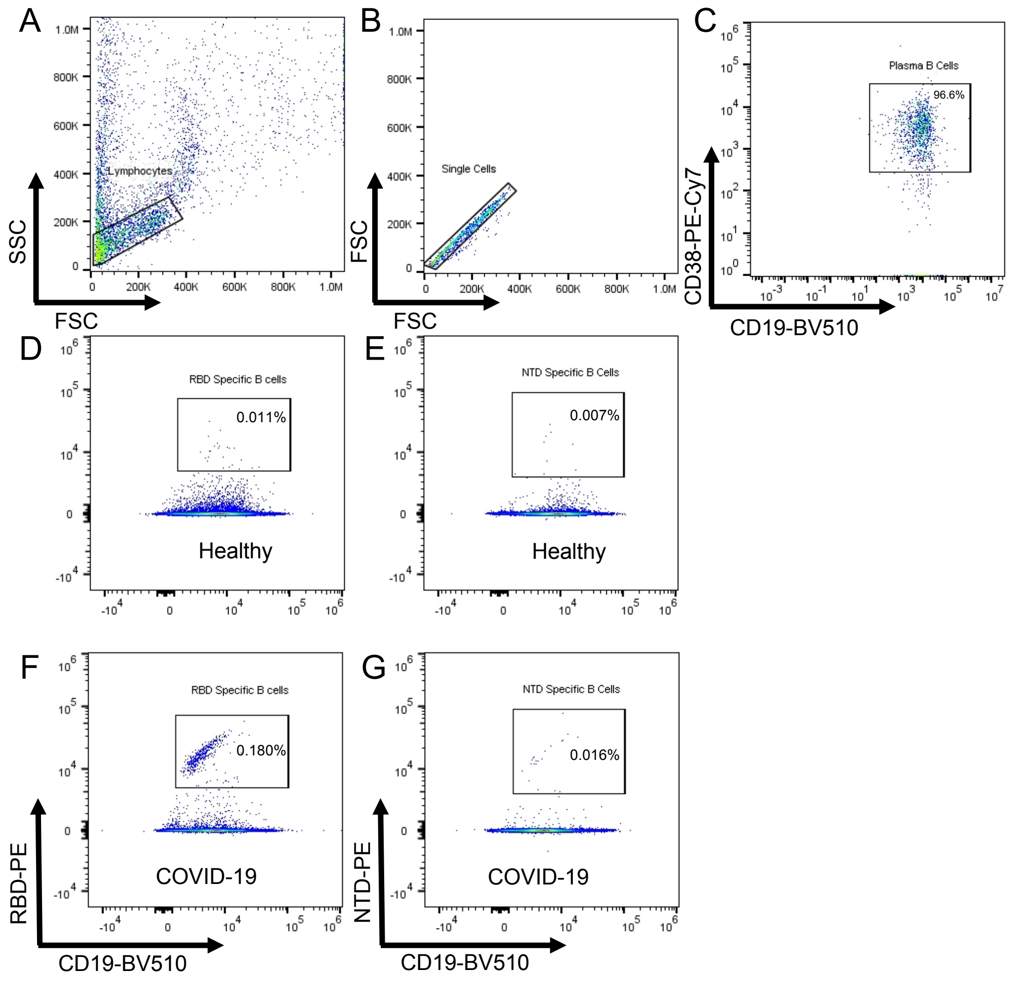

Figure S1. Gating strategy for CD38+ plasma B-cells and for RBD- or NTD-specific B-cells. Related to Figure 6. (A-C) Flow cytometry stainings of CD38+ plasma B-cells from patients infected with COVID-19 using two fluorescent markers, anti-human CD19-BV510 (BioLegend) and CD38-PE-Cy7 (BioLegend) in the same tube. Percentages indicate the proportions of $CD19^{+}CD38^{+}$ plasma B-cells within total B-cells. **(D-G)** Flow cytometry stainings of RBD- or NTD-specific B-cells from healthy donors and patients infected with COVID-19 using anti-human CD19-BV510 (BioLegend) and PE fluorescent dye for RBD protein (D, F) or NTD protein (E, G) in the same staining tube. Percentages indicate the proportions of $CD19^{+}$ and RBD- or NTD-protein double positive specific B cells.

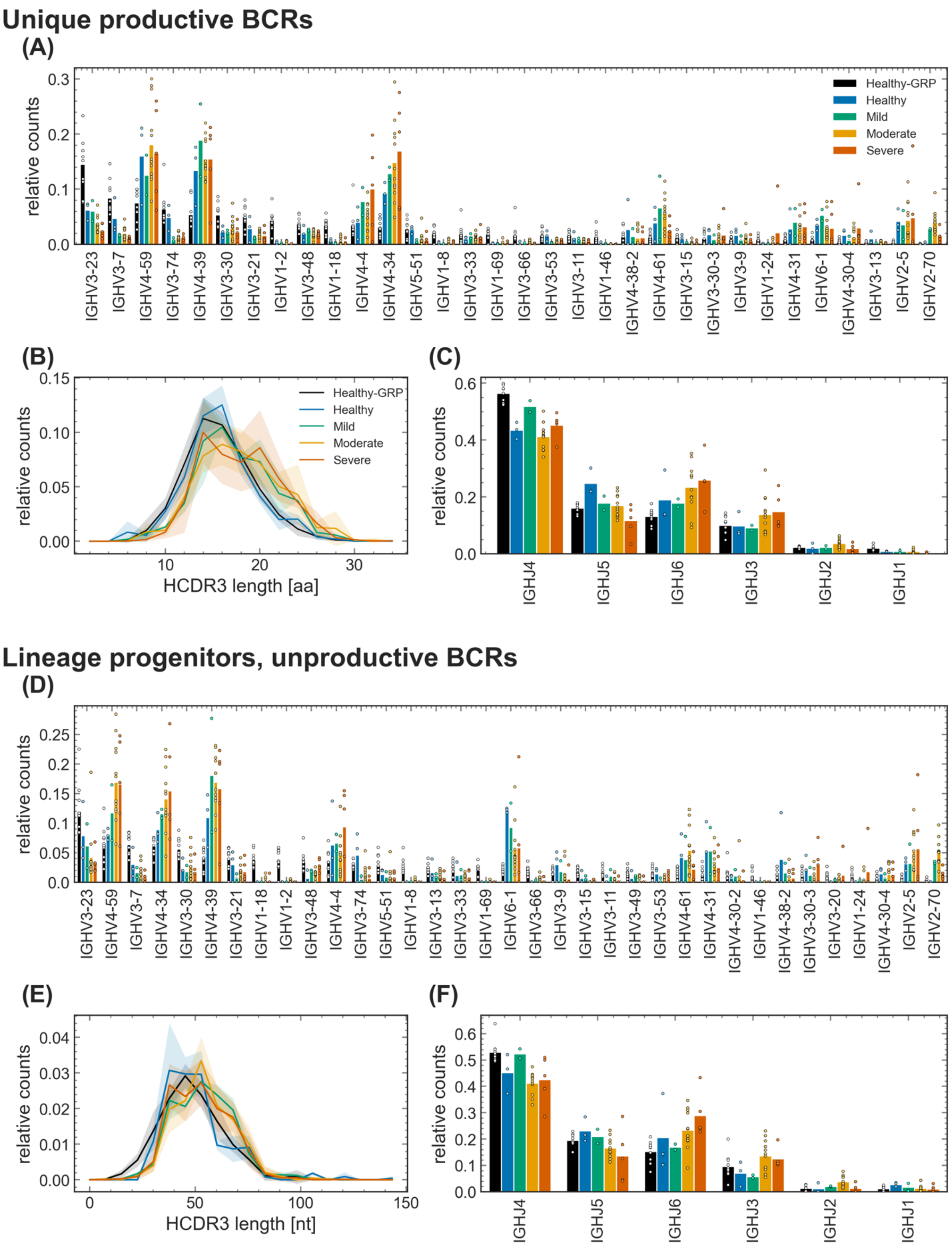

Unique productive BCRs
(A)
relative counts
0.0
0.1
0.2
0.3
Healthy-GRP
Healthy
Mild
Moderate
Severe
IGHV3-23
IGHV3-7
IGHV4-59
IGHV3-74
IGHV4-39
IGHV3-30
IGHV3-21
IGHV1-2
IGHV3-48
IGHV1-18
IGHV4-4
IGHV4-34
IGHV5-51
IGHV1-8
IGHV3-33
IGHV1-69
IGHV3-66
IGHV3-53
IGHV3-11
IGHV1-46
IGHV4-38-2
IGHV4-61
IGHV3-15
IGHV3-30-3
IGHV3-9
IGHV1-24
IGHV4-31
IGHV6-1
IGHV4-30-4
IGHV3-13
IGHV2-5
IGHV2-70
(B)
relative counts
0.00
0.05
0.10
0.15
Healthy-GRP
Healthy
Mild
Moderate
Severe
10
20
30
HCDR3 length [aa]
(C)
relative counts
0.0
0.2
0.4
0.6
IGHJ4
IGHJ5
IGHJ6
IGHJ3
IGHJ2
IGHJ1
Lineage progenitors, unproductive BCRs
(D)
relative counts
0.0
0.05
0.1
0.15
0.2
0.25
IGHV3-23
IGHV4-59
IGHV3-7
IGHV4-34
IGHV3-30
IGHV4-39
IGHV3-21
IGHV1-18
IGHV1-2
IGHV3-48
IGHV4-4
IGHV3-74
IGHV5-51
IGHV1-8
IGHV3-13
IGHV3-33
IGHV1-69
IGHV6-1
IGHV3-66
IGHV3-9
IGHV3-15
IGHV3-11
IGHV3-49
IGHV3-53
IGHV4-61
IGHV4-31
IGHV4-30-2
IGHV1-46
IGHV4-38-2
IGHV3-30-3
IGHV3-20
IGHV1-24
IGHV4-30-4
IGHV2-5
IGHV2-70
(E)
relative counts
0.00
0.01
0.02
0.03
0.04
0
50
100
150
HCDR3 length [nt]
(F)
relative counts
0.0
0.1
0.2
0.3
0.4
0.5
0.6
IGHJ4
IGHJ5
IGHJ6
IGHJ3
IGHJ2
IGHJ1

Figure S2. Bulk repertoire sequence statistics. Related to Figure 2. (A-C) Similar statistics are shown as in Fig. 2 (A, C-D), but for unique receptors excluding singletons (Methods). Unique BCRs in healthy individuals (our control and the Great Repertoire Project (GRP) by (Briney et al., 2019)) show significantly shorter HCDR3s compared to moderate and severe cohorts. ANOVA statistics for mean HCDR3 length between cohorts: Healthy-Mild: $F_{1,3} = 8.7$, p-value = 0.06; Healthy-Moderate: $F_{1,13} = 17.2$, p-value = 0.001; Healthy-Severe: $F_{1,6} = 10.0$, p-value = 0.020; GRP-Mild: $F_{1,10} = 11.3$, p-value = 0.0073; Healthy-GRP: $F_{1,11} = 0.074$, p-value = 0.791; GRP-Moderate: $F_{1,20} = 34.0$, p-value = 0.000011; GRP-Severe: $F_{1,13} = 41.5$, p-value = 0.000022. **(D-F)** Similar statistics are shown as in Fig. 2 (A, C-D), but for unproductive lineage progenitors. The differences in the statistics of HCDR3 length between the unproductive repertoires of healthy individuals and the COVID-19 cohorts are insignificant (ANOVA p-value > 0.01). Colors are consistent across panels. Detailed statistics on the biological and technical replicates can be found in Data S1.

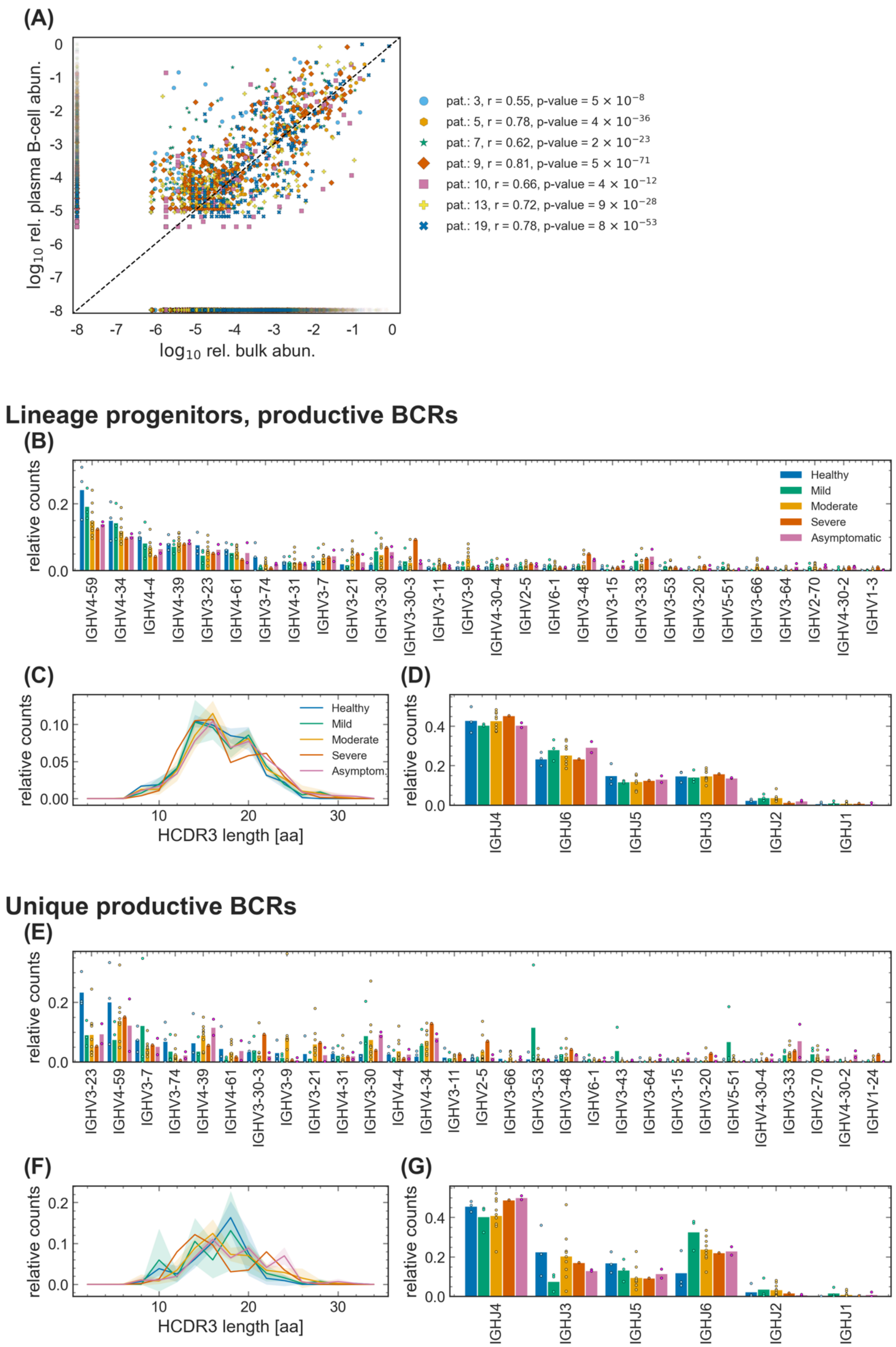
(A)
log10 rel. plasma B-cell abun.
log10 rel. bulk abun.
pat.: 3, r = 0.55, p-value = 5 × 10^−8
pat.: 5, r = 0.78, p-value = 4 × 10^−36
pat.: 7, r = 0.62, p-value = 2 × 10^−23
pat.: 9, r = 0.81, p-value = 5 × 10^−71
pat.: 10, r = 0.66, p-value = 4 × 10^−12
pat.: 13, r = 0.72, p-value = 9 × 10^−28
pat.: 19, r = 0.78, p-value = 8 × 10^−53
Lineage progenitors, productive BCRs
(B)
relative counts
Healthy
Mild
Moderate
Severe
Asymptomatic
IGHV4-59 IGHV4-34 IGHV4-4 IGHV4-39 IGHV3-23 IGHV4-61 IGHV3-74 IGHV4-31 IGHV3-7 IGHV3-21 IGHV3-30 IGHV3-30-3 IGHV3-11 IGHV3-9 IGHV4-30-4 IGHV2-5 IGHV6-1 IGHV3-48 IGHV3-15 IGHV3-33 IGHV3-53 IGHV3-20 IGHV5-51 IGHV3-66 IGHV3-64 IGHV2-70 IGHV4-30-2 IGHV1-3
(C)
relative counts
HCDR3 length [aa]
Healthy
Mild
Moderate
Severe
Asymptom.
(D)
relative counts
IGHJ4 IGHJ6 IGHJ5 IGHJ3 IGHJ2 IGHJ1
Unique productive BCRs
(E)
relative counts
IGHV3-23 IGHV4-59 IGHV3-7 IGHV3-74 IGHV4-39 IGHV4-61 IGHV3-30-3 IGHV3-9 IGHV3-21 IGHV4-31 IGHV3-30 IGHV4-4 IGHV4-34 IGHV3-11 IGHV2-5 IGHV3-66 IGHV3-53 IGHV3-48 IGHV6-1 IGHV3-43 IGHV3-64 IGHV3-15 IGHV3-20 IGHV5-51 IGHV4-30-4 IGHV3-33 IGHV2-70 IGHV4-30-2 IGHV1-24
(F)
relative counts
HCDR3 length [aa]
(G)
relative counts
IGHJ4 IGHJ3 IGHJ5 IGHJ6 IGHJ2 IGHJ1

Figure S3. Sequence features of immune receptors in the plasma B-cell repertoire across cohorts. Related to Figure 2. (A) Scatter plot shows $\log_{10}$ relative abundance of clonal lineages constructed from the plasma B-cell and bulk repertoire data from all time points and technical replicates in each patient (colors). To avoid primer-specific amplification biases, the relative abundance is estimated as the total read count of a clonal lineage relative to the total reads in the data associated with a specific primer amplification. Lineages with only bulk reads or only plasma reads are displayed as having $\log_{10}$ relative abundance = 1e-8. Pearson correlations (r) between abundances of lineages which were present in both the bulk and the plasma B-cell repertoires and the corresponding p-values are indicated in the legend for each patient. **(B-D)** Similar statistics are shown as in Fig. 2 (A,C,D), but for progenitors of clonal lineages with minimum size of three, in which at least one BCR is found in the plasma B-cell repertoire data; statistics of these lineages are reported in Data S1. Smaller read counts in the plasma B-cell data compared to the bulk do not allow for comparative analysis of receptor statistics across cohorts. Markers shown in the histograms indicate statistics for each individual (biological replicate) in a given cohort. **(E-G)** Similar statistics are shown as in Fig. S2 (A-C), but for unique receptors harvested from the plasma B-cell repertoires. Statistics of these receptors for each individual is described in Data S1. Smaller read counts in the plasma B-cell data compared to the bulk don't allow for comparative analysis of receptor statistics across cohorts. Colors are consistent across panels.

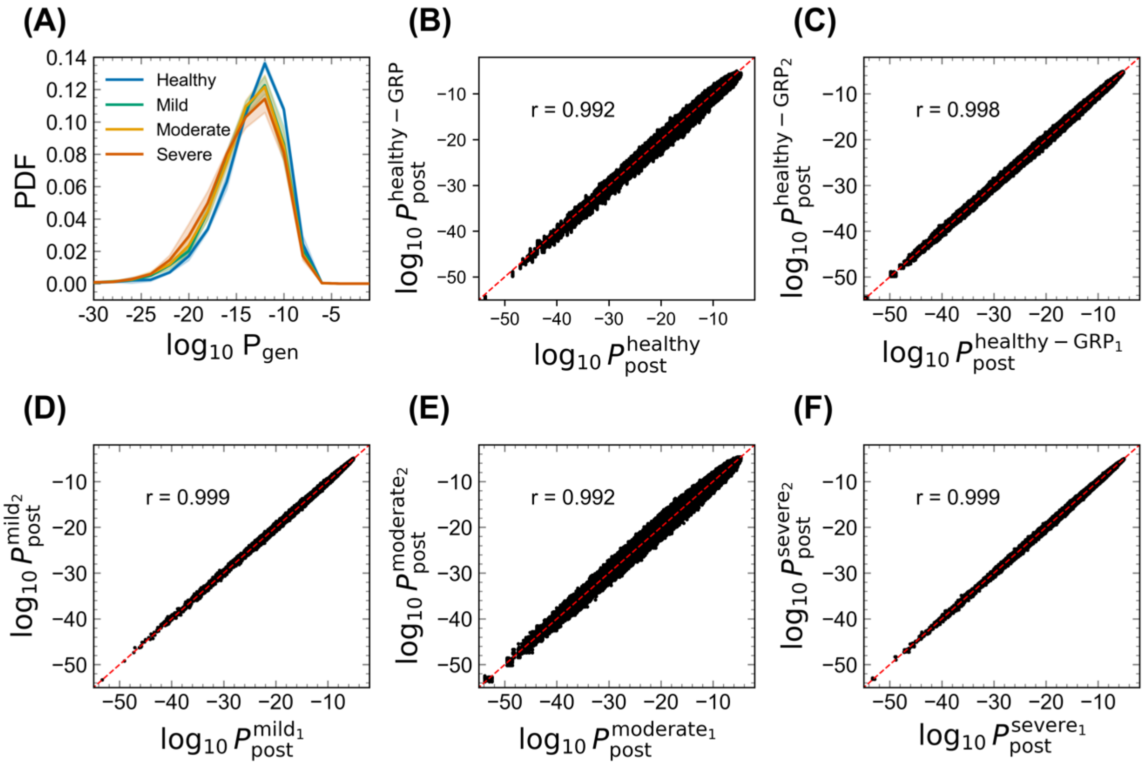

Figure S4. Robustness of SONIA selection models. Related to Figure 3. (A) The distribution of the log-generation probability of a sequence σ $\log_{10} \mathrm{P}_{\mathrm{gen}}(\sigma)$, evaluated using the inferred generation models by the IGoR software (Marcou et al., 2018), is shown as a normalized probability density function (PDF) for inferred naïve progenitors of productive clonal lineages in cohorts of healthy individuals and the mild, moderate, and severe cohorts of patients with COVID-19 (colors). Full lines show distributions averaged over individuals in each cohort, and shadings indicate regions containing one standard deviation of variation among individuals (biological replicates) within a cohort. **(B)** The scatterplot shows log P_{post} obtained by evaluating 500,000 generated sequences using the inferred selection (SONIA) models (Sethna et al., 2020) trained on the healthy cohort (x-axis) and 30 SONIA models trained on independent samples of the GRP dataset (Briney et al., 2019) down-sampled to the size of the healthy cohort in this study (7,161 receptors) (y-axis). The scatterplots show all unique pairwise comparisons between SONIA models trained on independent subsets with each cohort for **(C)** GRP (30 models), and patients with COVID-19 with **(D)** mild (two models), **(E)** moderate (13 models), and **(F)** severe (three models) symptoms (Methods). The Pearson correlation for all pairwise model comparisons is shown in each panel. Detailed statistics on the biological replicates can be found in Data S1.

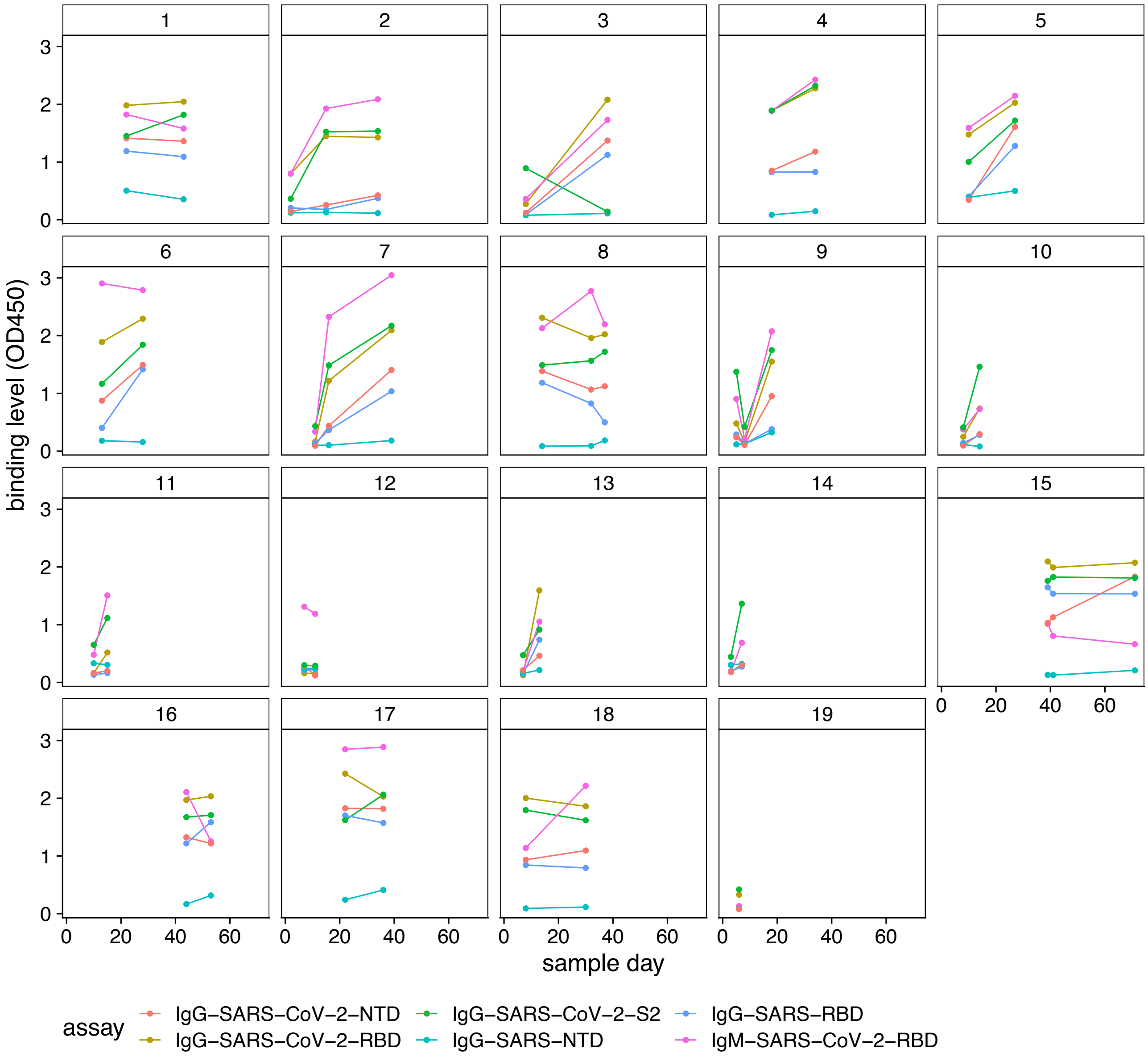

Figure S5. ELISA binding assays for IgG and IgM repertoires against SARS-CoV-2 and SARS-CoV. Related to Figure 4. Plasma binding levels (measured by OD_{450} in ELISA) against RBD, NTD, and S2 subdomain of SARS-CoV-2 and against RBD and NTD epitopes of SARS-CoV are shown. As seen in binding assays, many individuals developed a cross-reactive response to SARS-CoV-2 and SARS-CoV. Some individuals showed no increase in IgG binding to SARS-CoV-2 RBD due to already high levels at sampling time or natural variation. For the expansion analysis (Fig. 4), we only analyzed individuals whose IgG repertoires showed an increase in binding to SARS-CoV-2 (RBD): 2, 3, 4, 5, 6, 7, 9, 10, 11, 13, and 14.

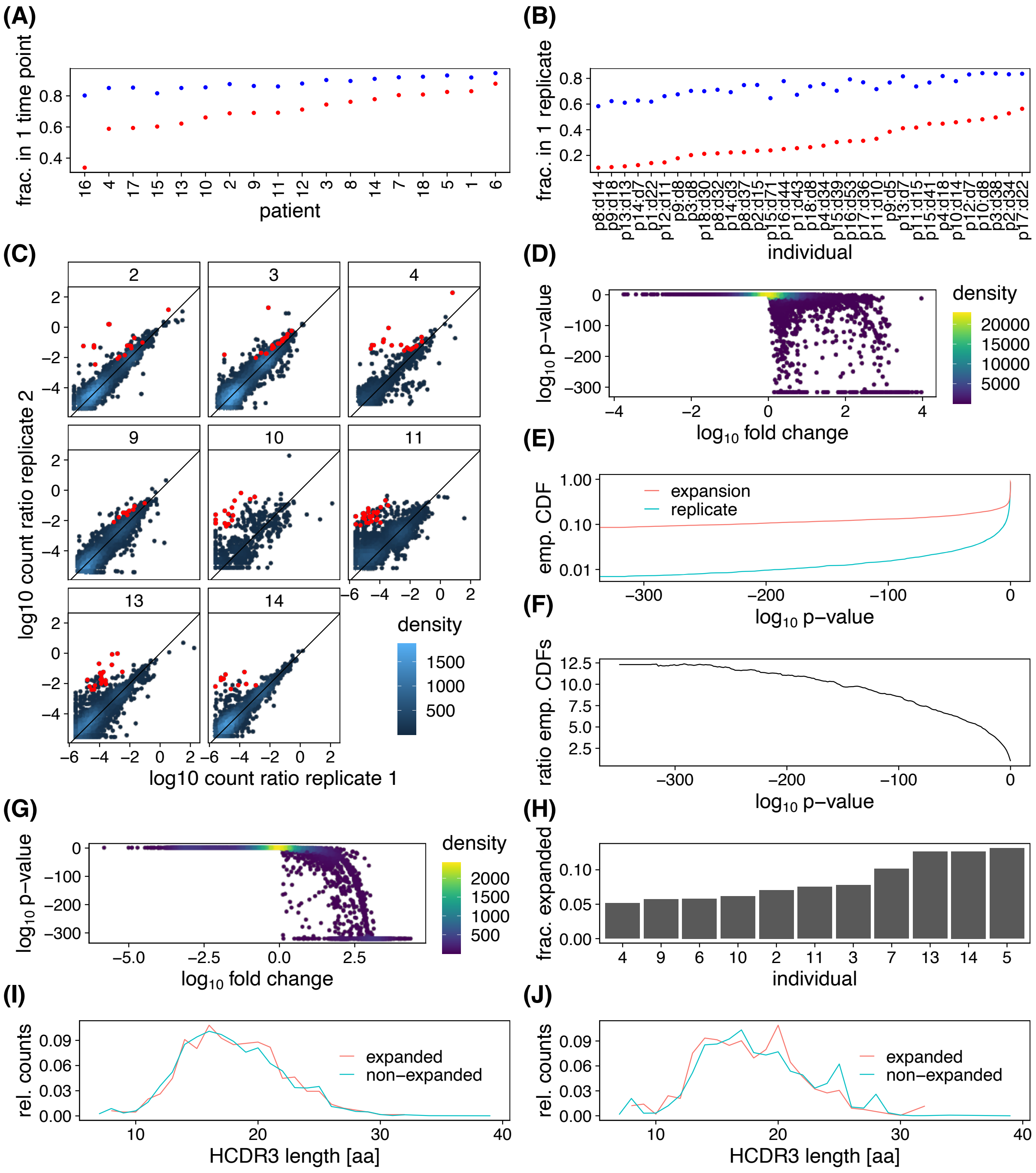

Figure S6. Statistics of clonal expansion. Related to Figure 4. BCR repertoires are highly undersampled, and relatively few BCR lineages appear in multiple time points and technical replicates. **(A)** Fraction of lineages present in only one time point before (blue) and after (red) filtering out small lineages (i.e., those with less than three unique sequences per time point) are shown. **(B)** Fraction of

lineages present in only one technical replicate before (blue) and after (red) filtering out small lineages (i.e., those with less than three unique sequences per time point) are shown. **(C)** The log-ratio of abundance of receptors for all clonal lineages present in two technical replicates is shown. Each panel shows the test result for a given patient, as indicated in the label. The count density indicates the number of lineages at each point. Lineages that show a significant expansion over time are indicated in red. Since this is technical replicate data and represents a null model, red points indicate false positives. **(D)** $\log_{10}$ p-values of the expansion test versus $\log_{10}$ fold change (or odds ratio) for the technical replicate data are shown. Color indicates density of points, and p-values of zero are displayed at the minimum nonzero value. See Methods for normalization, data processing, and hypothesis testing. **(E)** Empirical cumulative density functions (CDF) of expansion data from multiple time points (red) and the technical replicate data (blue) show that many more tests performed on multiple time point data result in low p-values compared to the technical replicate data. **(F)** Ratio of empirical cumulative density functions (CDF) indicates that at a significance threshold of 10^{-300} there are roughly 12.3 times more true positives than false positives. **(G)** $\log_{10}$ p-values of the expansion test versus $\log_{10}$ fold change (or odds ratio) for data corresponding to Fig. 4B is shown. Color indicates density of points, and p-values of zero are displayed at the minimum nonzero value. See Methods for normalization, data processing, and hypothesis testing. **(H)** Fraction of lineages expanded for different individuals is shown. HCDR3 length distributions of expanded and non-expanded lineages, **(I)** with each lineage having equal weight, and **(J)** with each lineage weighted by the number of unique sequences per time point (excluding singletons) are shown. Detailed statistics on the biological and technical replicates can be found in Data S1.

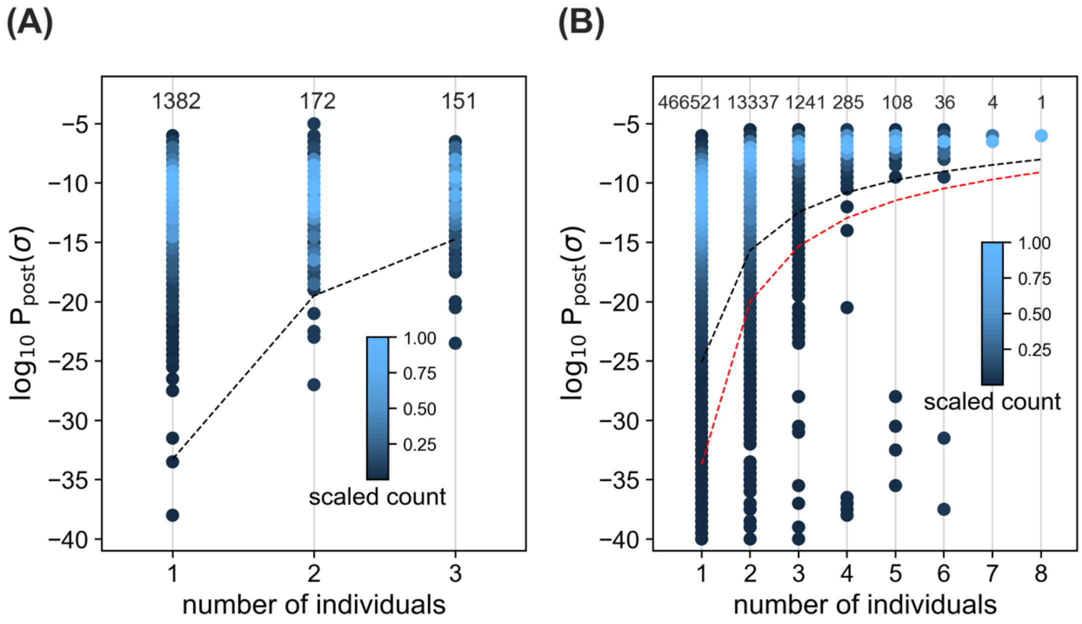

Figure S7. Sharing of BCRs among healthy individuals. Related to Figure 5. (A) The density plot shows the distribution of $\log_{10} P_{\text{post}}$ for progenitors of clonal lineages shared in a given number of healthy individuals, indicated on the horizontal axis; histogram bin size is 0.5. The clonal lineages are constructed from the bulk data (Tables S1). The counts in each bin are scaled such that the maximum is equal to one for each column. The numbers above each column indicate the total number of sequences in the respective column. Sharing of rare lineages with $\log_{10} P_{\text{post}}$ below the dashed line is statistically significant (see Methods). **(B)** Similar statistics as in **(A)** are shown but for healthy individuals in the Great Repertoire Project (Briney et al., 2019). Sharing of rare lineages with $\log_{10} P_{\text{post}}$ below the black dashed line is statistically significant (see Methods). For comparison, the dashed line in **(A)** is shown as a red dashed line in **(B)** and extended to eight individuals.

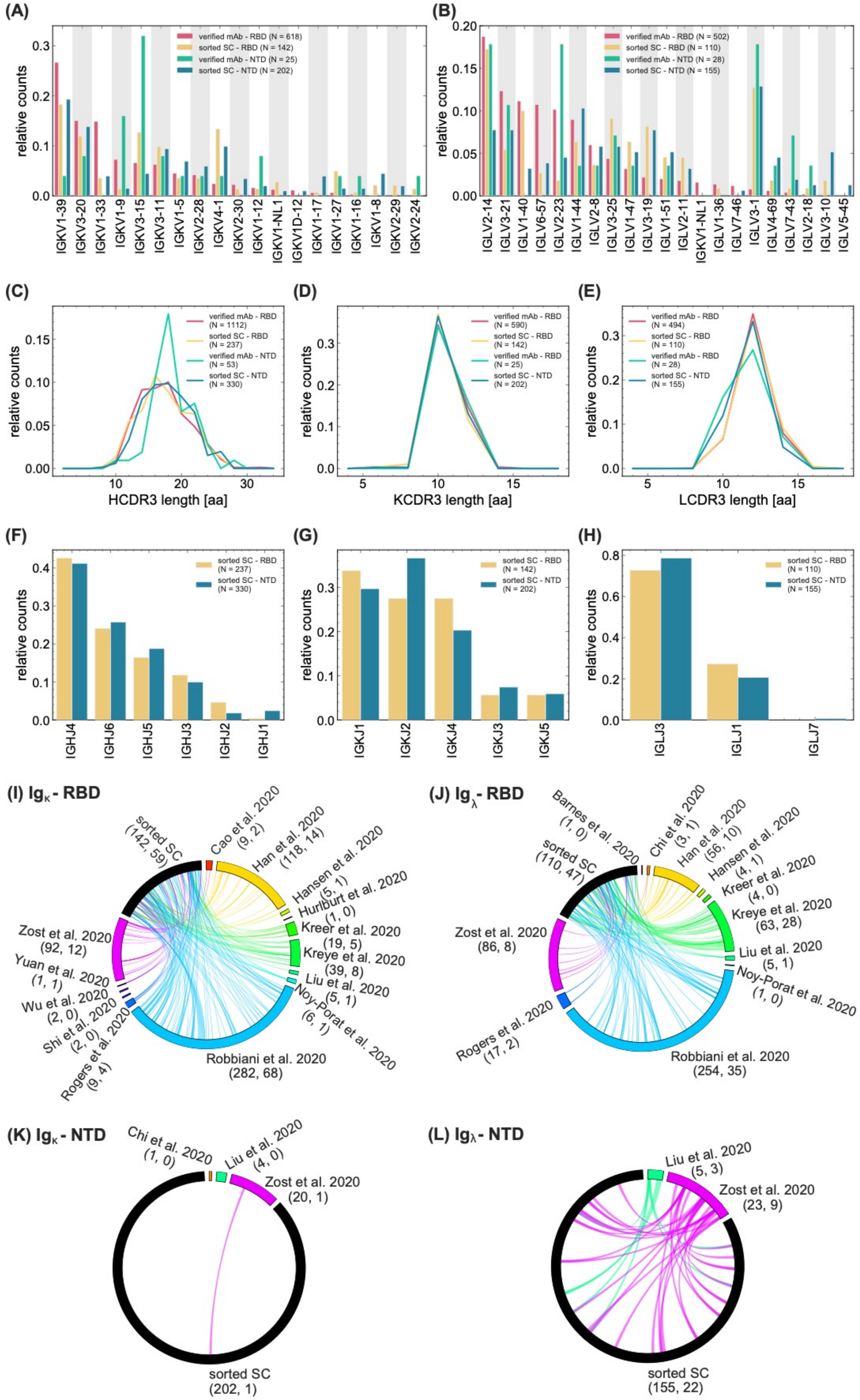

(A)
verified mAb - RBD (N = 618)
sorted SC - RBD (N = 142)
verified mAb - NTD (N = 25)
sorted SC - NTD (N = 202)
relative counts
IGKV1-39
IGKV3-20
IGKV1-33
IGKV1-9
IGKV3-15
IGKV3-11
IGKV1-5
IGKV2-28
IGKV4-1
IGKV2-30
IGKV1-12
IGKV1-NL1
IGKV1D-12
IGKV1-17
IGKV1-27
IGKV1-16
IGKV1-8
IGKV2-29
IGKV2-24
(B)
verified mAb - RBD (N = 502)
sorted SC - RBD (N = 110)
verified mAb - NTD (N = 28)
sorted SC - NTD (N = 155)
relative counts
IGLV2-14
IGLV3-21
IGLV1-40
IGLV6-57
IGLV2-23
IGLV1-44
IGLV2-8
IGLV3-25
IGLV1-47
IGLV3-19
IGLV1-51
IGLV2-11
IGKV1-NL1
IGLV1-36
IGLV7-46
IGLV3-1
IGLV4-69
IGLV7-43
IGLV2-18
IGLV3-10
IGLV5-45
(C)
verified mAb - RBD (N = 1112)
sorted SC - RBD (N = 237)
verified mAb - NTD (N = 53)
sorted SC - NTD (N = 330)
relative counts
HCDR3 length [aa]
(D)
verified mAb - RBD (N = 590)
sorted SC - RBD (N = 142)
verified mAb - RBD (N = 25)
sorted SC - NTD (N = 202)
relative counts
KCDR3 length [aa]
(E)
verified mAb - RBD (N = 494)
sorted SC - RBD (N = 110)
verified mAb - RBD (N = 28)
sorted SC - NTD (N = 155)
relative counts
LCDR3 length [aa]
(F)
sorted SC - RBD (N = 237)
sorted SC - NTD (N = 330)
relative counts
IGHJ4
IGHJ6
IGHJ5
IGHJ3
IGHJ2
IGHJ1
(G)
sorted SC - RBD (N = 142)
sorted SC - NTD (N = 202)
relative counts
IGKJ1
IGKJ2
IGKJ4
IGKJ3
IGKJ5
(H)
sorted SC - RBD (N = 110)
sorted SC - NTD (N = 155)
relative counts
IGLJ3
IGLJ1
IGLJ7
(I) Igκ - RBD
sorted SC (142, 59)
Cao et al. 2020 (9, 2)
Han et al. 2020 (118, 14)
Hansen et al. 2020 (5, 1)
Hurlburt et al. 2020 (1, 0)
Kreer et al. 2020 (19, 5)
Kreye et al. 2020 (39, 8)
Liu et al. 2020 (5, 1)
Noy-Porat et al. 2020 (6, 1)
Robbiani et al. 2020 (282, 68)
Rogers et al. 2020 (9, 4)
Shi et al. 2020 (2, 0)
Wu et al. 2020 (2, 0)
Yuan et al. 2020 (1, 1)
Zost et al. 2020 (92, 12)
(J) Igλ - RBD
Barnes et al. 2020 (1, 0)
sorted SC (110, 47)
Chi et al. 2020 (3, 1)
Han et al. 2020 (56, 10)
Hansen et al. 2020 (4, 1)
Kreer et al. 2020 (4, 0)
Kreye et al. 2020 (63, 28)
Liu et al. 2020 (5, 1)
Noy-Porat et al. 2020 (1, 0)
Robbiani et al. 2020 (254, 35)
Rogers et al. 2020 (17, 2)
Zost et al. 2020 (86, 8)
(K) Igκ - NTD
Chi et al. 2020 (1, 0)
Liu et al. 2020 (4, 0)
Zost et al. 2020 (20, 1)
sorted SC (202, 1)
(L) Igλ - NTD
Liu et al. 2020 (5, 3)
Zost et al. 2020 (23, 9)
sorted SC (155, 22)

Figure S8. Sequence features of heavy and light chain receptors in sorted single-cells and monoclonal antibodies. Related to Figure 6. The bar graphs show the relative counts for **(A)** the $\kappa -$ chain IGKV-gene usage and **(B)** the $\lambda -$ chain IGLV-gene usage for the verified mAbs reactive to RBD (pink) and NTD (green) epitopes of SARS-CoV-2 (Data S3) and the light chain receptors obtained from the RBD- (yellow) and NTD- (blue) sorted single cell data (Methods). Distributions of the lengths of **(C)** HCDR3 (heavy chain), **(D)** KCDR3 ($\kappa -$ chain), and **(E)** LCDR3 ($\lambda -$ chain) amino acid sequences are shown. **(F)** IGHJ-gene usage, **(G)** IGKJ-gene usage, and **(H)** IGLJ-gene usage of the sorted single-cells is shown in relative counts in bar graphs. Colors are consistent between panels and the number of samples used to evaluate the statistics in each panel is indicated in the legend. **(I-L)** Circos plots show matches between the light chain CDR3 sequences of progenitors in the sorted single-cell dataset (black) and light chain CDR3 sequences in the verified mAbs (colors) for RBD-reactive **(I)** IG_{κ} and **(J)** IG_{λ} sequences, and for NTD-reactive **(K)** IG_{κ} and **(L)** IG_{λ} sequences. Different colors indicate different studies from which mAbs were pooled. The reference to each study, the total number of mAbs in the study, and the number of mAbs with matching light chain CDR3 to the sorted single-cell data are reported in each panel.

5'-end primer	Sequence (5'-3')
IGHV1	CCTCAGTGAAGGTCTCCTGCAAGG
IGHV2	TCCTGCGCTGGTGAAACCCACACA
IGHV3	GGTCCCTGAGACTCTCCTGTGCA
IGHV4	TCGGAGACCCTGTCCCTCACCTGC
IGHV5	CAGTCTGGAGCAGAGGTGAAA
IGHV6	CCTGTGCCATCTCCGGGGACAGTG
**3'-end primer**	**Sequence (5'-3')**
CHG-R	GCGCCTGAGTTCCACGACAC

Table S1. List of primers used for PCR amplification of B-cell repertoires samples. Related to Figure 2.
